# Phenanthrene sorption studies on coffee waste– and diatomaceous earth–based adsorbents, and adsorbent regeneration with cold atmospheric plasma

**DOI:** 10.1007/s11356-023-27381-8

**Published:** 2023-05-11

**Authors:** Anastasia Stavrinou, Maria A. Theodoropoulou, Christos A. Aggelopoulos, Christos D. Tsakiroglou

**Affiliations:** 1 Institute of Chemical Engineering Sciences, Foundation for Research and Technology Hellas, Stadiou Str, Platani, 26504 Patras, Greece; 2 Department of Physics, University of Patras, 26504 Patras, Greece; 3 Hellenic Open University, 26335 Patras, Greece

**Keywords:** Adsorption, PAH, Activated carbon, Coffee waste, Pore structure, Diffusion, Mass transfer, Cold atmospheric plasma

## Abstract

Phenanthrene (PHE) is a polycyclic aromatic hydrocarbon categorized as a high priority organic pollutant being toxic for the ecosystem and human health, and its sorption on natural organic or inorganic substances seems a well-promising method for its removal from water streams. The goals of the present work are (i) to assess the capacity of low-cost adsorbents fabricated by treating coffee wastes and diatomaceous earth to remove PHE from water; (ii) to elucidate the role of the pore structure on PHE sorption dynamics; and (iii) to assess the potential to regenerate adsorbents loaded with PHE, by using the novel technology of cold atmospheric plasma (CAP). Diatomaceous earth (DE) and DE pre-treated with sodium hydroxide (NaOH) or phosphoric acid (H_3_PO_4_) were chosen as inorganic adsorbents. Coffee waste (CW) and activated carbons (AC) produced from its pyrolysis at 800 °C (CWAC), either untreated (CWAC-800) or pre-treated with NaOH (CWAC-NaOH-800) and H_3_PO_4_ (CWAC-H_3_PO_4_-800), were chosen as organic adsorbents. The adsorbents were characterized with nitrogen adsorption–desorption isotherms, attenuated total reflectance-Fourier transform infrared spectroscopy, Raman spectroscopy, scanning electron microscopy, and mercury intrusion porosimetry. Based on the PHE sorption capacity and pore structure/surface characteristics, the CWAC-NaOH-800 was chosen as the most efficient adsorbent for further equilibrium and kinetic sorption studies. The multi-compartment model was used to describe the PHE sorption dynamics in CWAC-NaOH-800 by accounting for the pore/surface diffusion and instantaneous sorption. The CWAC-NaOH-800 exhibited remarkable values for (i) the specific surface area (*S*_BET_ = 676.5 m^2^/g) and meso- and micro-pore volume determined by nitrogen sorption (*V*_LN2_ = 0.415 cm^3^/g); (ii) the macro- and meso-pore volume determined by mercury intrusion porosimetry (*V*_MIP_ = 3.134 cm^3^/g); and (iii) the maximum PHE sorption capacity (*q*_max_ = 142 mg/g). The percentage of adsorbent recovery after its regeneration with CAP was found to be ~ 35%. From the simulation of sorption dynamics, it was found that at early times, the sorption kinetics is governed by the film diffusion towards the external surface of grains, but at late times, most of the adsorbed mass is transferred primarily to meso-/macro-pores via diffusion, and secondarily to micro-porosity via surface diffusion. Based on the adsorbent characteristics, effect of pH on sorption efficiency, and numerical analysis of sorption dynamics, it was concluded that probably the dominant adsorption mechanism is the π-π interactions between hydrophobic PHE aromatic rings and CWAC-NaOH-800 graphene layers. The high PHE removal efficiency of CWAC-NaOH-800, the successful interpretation of sorption dynamics with the multi-compartment model, and the potential to regenerate PHE-loaded adsorbents with the green and economic technology of CAP motivate a strategy for testing CWACs towards the adsorption of other PAHs, application of adsorbents to real wastewaters, and scaling-up to pilot units.

**Graphical Abstract:**

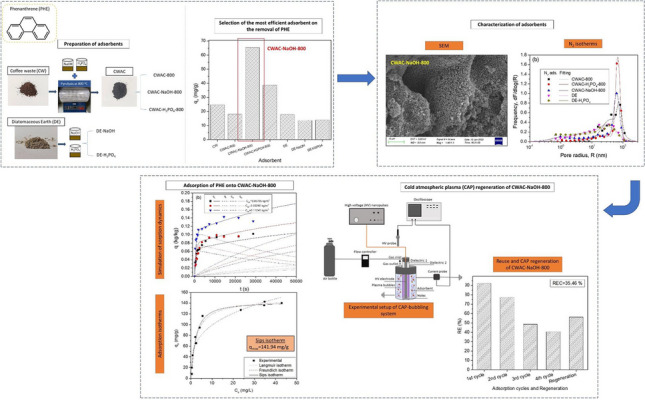

## Introduction

Polycyclic aromatic hydrocarbons (PAHs) are recalcitrant pollutants characterized by low biodegradability and water solubility being very harmful for the aquatic flora and fauna, and carcinogenic and mutagenic (Kaya et al. 2013; Gupta and Singh 2018; Fabian et al. 2022). For these reasons, the US Environmental Protection Agency (USEPA) and European Community Water Framework Directive have classified PAHs to the high priority pollutants (EPA 1980; European Commission 2014). PAHs are commonly released from multiple sources during incomplete combustion. Some examples are waste incineration, iron production and cement industry, coal-tar pitch production, dye manufacturing, pesticide production, refineries, power plants, vehicle exhaust gases, garbage burning, coal coking, wood burning, cooking in oil/gas burners, and agricultural waste burning. Atmospheric PAHs are deposited in surface water, soil, and plants through dry/wet deposition processes, and gradually, they are transferred to the groundwater, plants, and food (Patel et al. 2020).

Phenanthrene (PHE) belongs to the USEPA priority PAH list; it is a hydrophobic aromatic hydrocarbon with three aromatic rings, detected very frequently at high concentrations in water, air, and soil. It is lipophilic, which makes easy its adsorption on the gastrointestinal tract and transfer to the lungs (Ifegwu and Anyakora 2016; Pedetta et al. 2013). There is a variety of methods used for the removal of PHE from wastewater and soil. In general, bioremediation and phytoremediation are used widely for the degradation of PAHs (Pourfadakari et al. 2021). Wang et al. (2023) developed biochar-immobilized bacterial microspheres for the removal of PHE from water and soil through bioremediation. Phytoremediation has been used for PHE removal from soil (Khan et al. 2014). Although biological methods evolve the last years and new techniques are discovered, they are characterized by some disadvantages concerning the remediation of PAHs: the limited bioavailability of PAHs, the low abundance, diversity, and activity of indigenous hydrocarbon-degrading bacteria, and their slow growth rates (Kronenberg et al. 2017). Membrane filtration, advanced oxidation processes (AOPs), and adsorption have been reported as physical–chemical methods for the removal of PAHs (Gutierrez-Urbano et al. 2021). Regarding the use of AOPs, Dai et al. (2022) synthesized plasmonic Ag/Ag_3_PO_4_/g-C_3_N_4_ heterojunction photocatalyst to degrade PHE from water, and Treviño Reséndez and Mijaylova (2021) have reported the removal of naphthalene and PHE by electro-oxidation coupled with membrane bioreactor. Despite the very interesting advances done on a variety of physicochemical methods, there are still limitations concerning the high operating costs, the generation of secondary environmental contaminants, and the low removal efficiencies (Kubra et al. 2021a, c).

Among the existing techniques for wastewater treatment, adsorption is considered superior due to its cost-effectiveness, simple operation, high efficiency, and selectivity towards a wide range of pollutants. In addition, it provides a stable complexation mechanism of adsorbate-adsorbent system, because of the high concentration factor values based on the functional group attachments (Awual 2017; Kubra et al. 2021b). Various kinds of adsorbents have been reported in the literature for the removal of different pollutants, such as raw agricultural waste (AW) like banana, potato, and cucumber peels (Stavrinou et al. 2018), and coffee waste (Anastopoulos et al. 2017); activated carbon (AC) produced from (AW) such as banana peel AC (Stavrinou et al. 2023), coconut shell AC (Chan et al. 2022), and rice husk (Menya et al. 2018); natural inorganic compounds (NIC) such as zeolites (Díez et al. 2023) and diatomaceous earth (DE) (Yang et al. 2017); polymers such as biodegradable polymeric bioadsorbents (Kubra et al. 2021c) (Hasan et al. 2021); and novel emerging materials such as a broad variety of ligand based composites (Kubra et al. 2021a; Awual 2015, 2017, 2019a, b, c; Salman et al. 2021; Awual et al. 2015). AW and NIC are abundant in nature and costless and their utilization is an important step towards economic and environmental sustainability. Nevertheless, very often they have low adsorption capacity, as their selection depends on the type of the pollutant. As a result, they usually need some type of modification to increase their efficiency. The most common way to enhance the adsorption capacity is the pre-treatment with chemicals and/or production of AC with pyrolysis of AW. Regarding the activation process, metal salts (zinc chloride, ZnCl_2_), alkaline salts (potassium hydroxide, KOH; sodium hydroxide, NaOH), and acids (sulfuric acid, H_2_SO_4_; phosphoric acid, H_3_PO_4_) have been used as activating agents (Islam et al. 2023). Among them, H_3_PO_4_ is widely used due to the synthesis of meso-porosity, resulting in high total pore volume and pore diameters (Neme et al. 2022). NaOH is an excellent activating agent to be used at the pre-treatment stage of AC production as it creates a rugged adsorbent surface with different pore sizes, leading to a well-developed pore network. In addition, it is a cheaper and more environmental-friendly material compared to other chemicals (Pezoti et al. 2016). AC is commonly characterized by high specific surface area, porosity, and presence of functional groups. Low-cost AC produced from AW can be a very attractive and versatile adsorbent, given that the energy cost for activation, regeneration, and synthesis, as well as the environmental fingerprint, associated with the emission of nitrogen oxides (NO_x_), carbon monoxide (CO), and volatile organic compounds (VOCs), is kept at low acceptable levels (Islam et al. 2021; Stavrinou et al. 2023). Concerning the removal of PHE from water, several types of adsorbents have been used, such as walnut shell activated carbon (Wu et al. 2020), carbon nanotubes (Hou et al. 2013), stevensite and sepiolite (González-Santamaría et al. 2017), and activated carbon (Wang et al. 2022).

Coffee is the most popular beverage worldwide with a production of about 6 million tones/year, with the volume of husk, bean, and spent ground residues being enormous. In the framework of sustainable economy, the coffee derivatives are utilized for bio-energy production, as fertilizers for composting, as nutraceuticals, and as low-cost adsorbents (McNutt and He 2018). Coffee wastes (CW) are lignocellulosic materials with a complex composition of valuable elements and very good physicochemical properties which make them very good candidates to be used as adsorbents for various types of pollutants. The hydroxyl, carboxyl, and carbonyl groups of CW can interact with the molecules of pollutants which can be adsorbed on the surface. In addition, CW can be transformed to highly porous AC (CWAC) able to adsorb and attach a great variety of pollutants (Figueroa Campos et al. 2021). An important chemical property of ACs is their hydrophobicity (Bernal et al. 2018), which makes them good adsorbents for the removal of hydrophobic compounds like PHE. On the other hand, the disposal of coffee residues has undesirable environmental consequences, and its valorization has significant environmental and economic prospects. CW and CWAC have formerly been used as adsorbents for the removal of various organic and inorganic pollutants from water. Concerning the adsorption of heavy metals, Loulidi et al. (2021) utilized untreated CW to remove Cr (VI) and reached about 90% removal efficiency, and Edathil et al. (2018) produced magnetic CW nanocomposites for the adsorption of lead, achieving adsorption capacity equal to 41.15 mg/g. CW and its derivatives have been proved efficient for the adsorption of organic pollutants as well. Chen et al. (2022) used CWAC of high surface area, equal to 952.7 m^2^/g, to remove rhodamine B dye and achieved the high uptake capacity of 83.4 mg/g. Hgeig et al. (2019) prepared CWAC pre-treated with H_3_PO_4_ for the efficient adsorption of the pesticides carbendazim and linuron from aqueous solutions, and Lee et al. (2021) used CWAC pre-treated with NaOH to remove herbicides and achieved high adsorption capacities. Regarding the removal of PAHs, CW-based biochar has been used for the removal of PAHs from air (Tala and Chantara 2019), but no attempt has ever been done to treat PHE-polluted wastewater with adsorbents produced from CW.

Diatomaceous earth (DE) is a siliceous biogenic sediment created from the fossils of tiny aquatic plants, called diatoms. DE is an emerging adsorbent and has a great potential due to its low cost and abundancy in nature; its very good physical and chemical properties, such as high porosity, relatively high surface area, and thermal and mechanical stability; and mainly hydroxyl functional groups which can participate in hydrogen bonding with the molecules of pollutants (Zhao et al. 2020; ElSayed 2018). There are several studies which have reported the use of DE as adsorbent for heavy metals, dyes, and other organic pollutants. ElSayed (2018) investigated the removal of heavy metals from aqueous solutions with the natural DE, attaining high uptake efficiency. Dinh Du and Danh (2021) used alkali-activated Vietnamese DE to remove the mixture of rhodamine B and methylene blue dyes. With the pre-treatment with NaOH, the surface area was increased from 55.4 to 77.8 m^2^/g and a high removal efficiency was achieved. Nefzi et al. (2018) explored the adsorption of the herbicides chlortoluron and isoproturon and found DE maximum adsorption capacities equal to 1000 and 250 mg/g respectively. The use of DE as sorbents for the removal of PAHs from water, at least to our knowledge, has never been reported. In general, there is still a lack of knowledge on the potential of DE towards recalcitrant or persistent pollutants and the ways to enhance its adsorption capacity should be investigated more thoroughly.

Although adsorption processes are very effective, the issue of the valorization or disposal of the saturated adsorbents and the need of eco-friendly and low-cost technologies for their regeneration is still a challenge. During the last decade, the cold atmospheric plasma (CAP) has been introduced as a novel green, efficient, and economic advanced oxidation process with a variety of applications including solid waste and wastewater management, material modification, medicine, and agriculture (Lin et al. 2022). The most common CAP technology is the dielectric barrier discharge (DBD) reactor which is characterized by the presence of one or more dielectric layers between electrodes. The interaction of the generated CAP with water molecules produces reactive species such as hydroxyl radicals (**·**OH), oxygen (O), and nitrate (NO_3_^–^) and the emission of UV–visible light along with the generation of shockwaves (Kasih 2017). The advantage of this method is the achievement of the desired surface properties of adsorbents without the use of chemicals or UV photocatalysis lamps (Iervolino et al. 2019). Regarding the modification of adsorbents, the application of CAP modifies the surface of the sample with the formation of acidic functional groups without affecting its properties, leading to increased adsorption capacity (Kaya et al. 2016). Several works have reported the efficiency of CAP on adsorbent activation/modification (Krochmalny et al. 2022; Wu et al. 2018). In the case of adsorbent regeneration, the reactive species obtained by CAP can remove the adsorbed organic substances and recover the adsorbent (Gupta et al. 2018). Wu et al. (2018) successfully used the DBD reactor for regenerating AC from walnut shell after copper adsorption, and Zhou et al. (2016) achieved almost 100% recovery of tea waste modified with CAP after methylene blue dye adsorption. Giannoulia et al. (2023) efficiently regenerated halloysite nanoclay loaded with mixture of the antibiotic enrofloxacin and methylene blue dye with a CAP microbubble reactor and achieved its activation during the regeneration process. Plasma technology has been also successfully used for the remediation of PΗΕ contaminated soil (Li et al. 2016).

In spite of the numerous studies focusing on the development of adsorbents from low-cost wastes for the removal of a great variety of inorganic and organic pollutants, there is still a gap of knowledge concerning the linkage of the pore space properties with the performance of adsorbents towards the removal of one or more pollutants from water streams. On the other hand, the potential to remove PHE from wastewater with adsorbents produced from CW or DE has never been investigated. Finally, the combination of adsorption with a regeneration method utilizing an innovative advanced oxidation method like CAP could be recommended as an alternative two-step strategy for the remediation of wastewater from PAHs. In the present work, we studied the removal of PHE from aqueous solutions in batch mode by using two types of adsorbents: (i) CW and AC produced from CW only with pyrolysis at 800 °C (CWAC-800), or chemically pre-treated with NaOH (CWAC-NaOH-800) and H_3_PO_4_ (CWAC-H_3_PO_4_-800); (ii) DE and DE pre-treated with NaOH (DE-NaOH) and H_3_PO_4_ (DE-H_3_PO_4_). The PHE sorption efficiency was measured, and correlated with the pore structure and surface properties to select the adsorbent with the optimal properties, which was the AC produced from CW pre-treated with NaOH and pyrolyzed at 800 °C (CWAC-NaOH-800). Then, equilibrium and kinetic sorption studies were conducted along with a sensitivity analysis to pH. After the completion of four PHE adsorption cycles, CWAC-NaOH-800 was regenerated with DBD plasma to assess the potential to recover the adsorbent sorption capacity. A multi-compartment model was developed to describe the PHE sorption dynamics on porous granular CWAC, by using the pore structure properties as input parameters. The model was used to simulate kinetic sorption tests and estimate the mass transfer coefficients governing the rates of external and internal diffusive processes over the various compartments (external surface, meso-/macro-pore region, micro-pore region) of adsorbent with inverse modeling.

## Materials and methods

### Preparation of solutions and adsorbents

PHE (C_14_H_10_) was of purity ≥ 98%; all stock solutions were prepared with ethanol and all working aqueous solutions with distilled water. The ethanol–water percentage was equal to 30% v/v, and the solutions were prepared with stirring for 10 min followed by sonication for 10 min at 30 °C, and kept in dark at 4 °C to avoid biodegradation. For pH adjustment, NaOH and nitric acid (HNO_3_) were used. All chemicals used in the present work were of analytical grade.

**Fig. 1:**
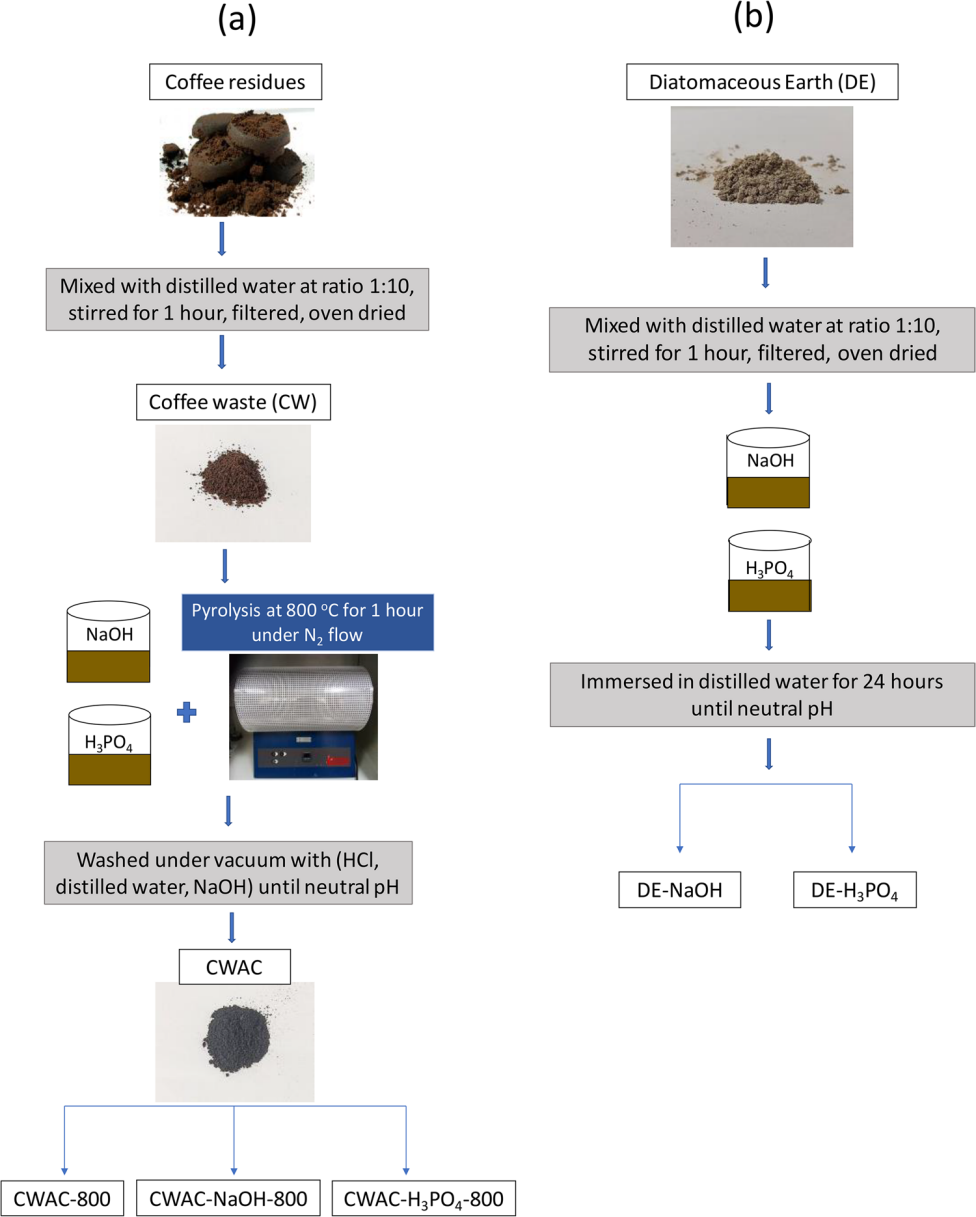
Flow chart of the preparation procedure of adsorbents. **a** CW, CWAC-800, CWAC-NaOH-800, CWAC-H_3_PO_4_-800. **b** DE, DE-NaOH, DE-H_3_PO_4_

CW was the residues of 100% Arabica espresso and was offered by a café situated in the University of Patras. DE was purchased from a local agricultural shop. The preparation procedure of adsorbents is presented in Fig. 1. Before chemical treatment, both adsorbents were mixed with distilled water in ratio 1:10, stirred for 1 h to remove the impurities, filtered, and oven-dried at 110 °C. Afterwards, CW and DE were immersed in NaOH or H_3_PO_4_ solution at ratio 1:1 for 16 h followed by filtering under a vacuum and oven-drying for 4 h. DE was immersed in distilled water for about 24 h until reaching neutral pH and obtaining the two new adsorbents, DE-NaOH and DE-H_3_PO_4_. Untreated CW, and CW after its chemical treatment with NaOH or H_3_PO_4_ were pyrolyzed in an annular oven (Lenton), at 800 °C at a rate of 10 °C/min, and kept at this temperature for 1 h, under the continuous flow of nitrogen at a rate of 1 L/min. Finally, the adsorbents were washed with hydrochloric acid (HCl) or NaOH and distilled water under vacuum until reaching neutral pH, and dried in an oven at 110 °C for 4 h. The activated carbons obtained by this procedure were named CWAC-800, CWAC-NaOH-800, and CWAC-H_3_PO_4_-800, respectively.

### Characterization of adsorbents and analytical techniques

The analysis of the pore structure of the adsorbents was accomplished with the N_2_ sorption isotherms which were measured at 77 K by using a TriStar 3000 V6.08 A sorption analyzer (Micrometrics). Prior to N_2_ sorption tests, the samples were dried and evacuated. The specific surface area, ${S}_{\mathrm{BET}}$, of the samples was calculated by applying the Brunauer–Emmett–Teller (BET) equation over low relative pressures of the N_2_ adsorption isotherm. The pore radius distribution was determined from N_2_ adsorption curve by using the Barrett, Joyner, and Halenda (BJH) method with the assumption that surface adsorption and capillary condensation occur in parallel, whereas the thickness of the adsorbed layer and the critical pore radius for liquid N_2_ condensation are governed by the Halsey and Kelvin equation, respectively (Tsakiroglou et al. 2021). The pore structure properties of CWAC-NaOH-800 were further analyzed over the macro-pore and meso-pore ranges, with mercury intrusion porosimetry (MIP) by using a computer-controlled Quantachrome PoreMaster 60 (Anton Parr).

The morphology of the adsorbents was examined by field-emission scanning electron microscopy (FE-SEM) (Zeiss SUPRA 35VP-FEG) operating at 5 or 10 keV. The functional groups of the surface of the adsorbents were identified with attenuated total reflectance-Fourier transform infrared spectroscopy (ATR-FTIR). FTIR spectral measurements were collected using a Thermo Scientific™ Nicolet™ iS20 FTIR Spectrometer with a diamond ATR crystal. All spectra were recorded in the range of 4000–400 cm^−1^, with an average of 34 scans and a spectral resolution of 0.25 cm^−1^ in transmittance mode. The chemical structure of the ACs derived from coffee waste, i.e., CWAC-800, CWAC-NaOH-800, and CWAC-H_3_PO_4_-800, was analyzed with Raman spectroscopy as well. For the Raman measurements, the T64000 Horiba Jobin Yvon micro-Raman setup was used. The excitation wavelength was 514.5 nm emitted from a DPSS laser (Cobolt Fandango TMISO laser, Norfolk, UK). The laser power on the sample was 1 mW. The backscattered radiation was collected from a single configuration of the monochromator after passing through an appropriate edge filter (LP02-633RU-25, laser2000, UK, Ltd., Huntingdon, Cambridgeshire, UK). The calibration of the instrument was achieved via the standard Raman peak position of Si at 520.5 cm^−1^. The spectral resolution was 5 cm^−1^.

The role of the surface charges of CWAC-NaOH-800 on the adsorption of PHE was determined by measuring the *ζ*-potential with a Malvern Nano-ZS Zetasizer. The following method was used (Niksirat et al. 2019): 0.01 g of CWAC-NaOH-800 was mixed with 100 mL sodium chloride 0.01 M. Afterwards, the pH of the suspension was adjusted over the range 2–10 with NaOH or HNO_3_, followed by sonication for 15 min. Finally, 0.8 mL of the suspensions was used for analysis.

The potential release of hazardous materials during the use of CWAC-NaOH-800 was evaluated by measuring the total organic carbon (TOC) content of distilled water equilibrated with the adsorbent. The suspension was shaken for 3 h on an overhead shaker and then centrifuged for 10 min at 10,000 rpm (Thermo Scientific, Heraeus Megafuge 16). The TOC of the supernatant was measured on an Analytik Jena TOC analyzer. The test was performed in duplicate, and the mean value of TOC was obtained.

### Adsorption experiments

The experiments were performed in glass bottles on an overhead shaker operating at a speed of 1.5 rpm and placed inside an incubator (Friocell) to keep a constant temperature at 25 °C. PHE solutions of initial concentration 30 mg/L were used to measure the sorption capacity of all adsorbents and select the most efficient one for further studies. Parametric analysis was carried out to examine the effect of initial pH (3–10), contact time (5–480 min), and initial PHE concentration (5–110 mg/L) on the sorption capacity of the selected adsorbent. In all experiments, 0.005 g of adsorbent was mixed with 10 mL of PHE solution. At the end of experiments, samples were collected from the bottles and centrifuged for 10 min at 10,000 rpm and the PHE concentration was measured with a UV–Visible spectrophotometer (Shimadzu UV-1900) by determining the characteristic peak at 251.50 nm on the UV-absorption spectrum. For the sake of accuracy, all experiments were conducted in duplicate. The amount of pollutant adsorbed at time $t$, ${q}_{t}$ (mg/g), and the adsorption capacity at equilibrium, ${q}_{e}$ (mg/g), were calculated by the following equations:

1 $${q}_{t}=\frac{{C}_{0}-{C}_{t}}{m}\times V$$

2 $${q}_{e}=\frac{{C}_{0}-{C}_{e}}{m}\times V$$

where ${C}_{0}$, ${C}_{t}$, and ${C}_{e }$(mg/L) are the PHE concentrations at time $t=0$, initially, time $t$, and equilibrium, respectively; $V$ is the volume of the PHE solution; and $m$ is the mass of the adsorbent.

### Cold atmospheric plasma–assisted regeneration

**Fig. 2:**
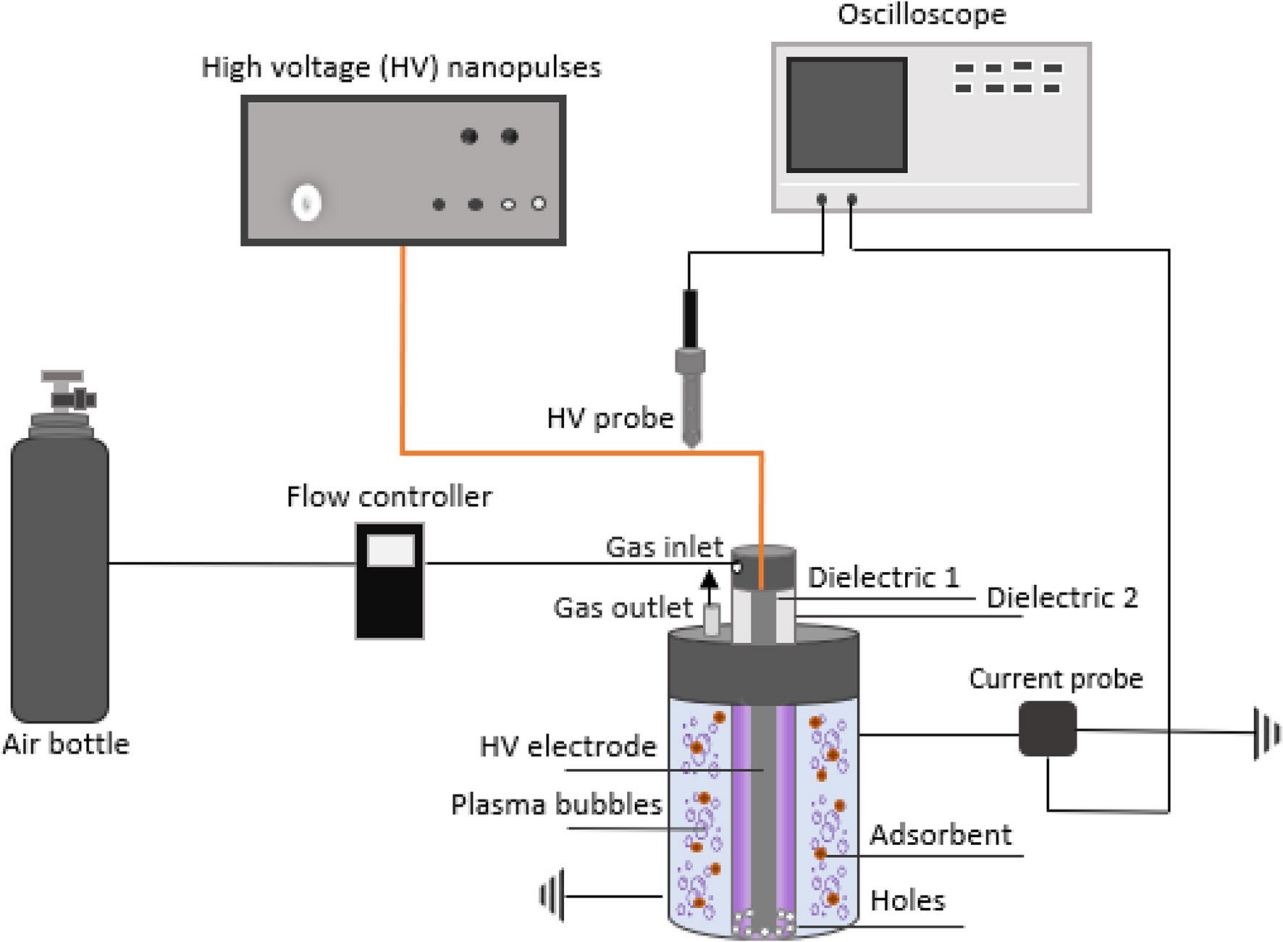
Schematic of the experimental setup of CAP-bubbling system

The regeneration of saturated adsorbent was conducted after having completed four (4) adsorption cycles, when the sorption capacity of adsorbent had been reduced by more than 50%. During the adsorption cycles, 10 mL of solutions containing 60 mg/L PHE was mixed with 0.005 g of adsorbent, in several bottles for a period of 180 min. Afterwards, 0.01 g of saturated adsorbent was mixed with 50 mL of deionized water and placed into a DBD plasma reactor to be treated for 30 min. The CAP experimental setup included a plasma microbubble reactor (CAP-bubbling), powered by a nanosecond pulse generator (NPG-18/3500) which produced positive high voltage nanopulses of very short rising time (~ 4 ns), and a discharge characterization system (Fig. 2). As presented in Fig. 2, the reactor consisted of a high voltage (HV) stainless-steel electrode placed inside an inner quartz tube (dielectric 1). An outer quartz tube (dielectric 2) forms a coaxial DBD where plasma phase was generated (Meropoulis and Aggelopoulos 2023). The plasma gas was injected into the space between the two dielectrics and inserted in the form of bubbles directly into the water through evenly spaced holes around the base of the outer tube. At the external surface of the reactor, a stainless-steel grid was attached acting as the grounded electrode. The pulse voltage was 33 kV, the frequency was set at 200 Hz, and air was injected at a flow rate of 3 L/min, controlled by a mass flow controller (Aalborg GFC17) (Giannoulia et al. 2023). After the regeneration stage, the adsorbent was tested with regard to the PHE adsorption, under conditions identical to those of earlier adsorption cycles. The recovery of sorption capacity, $\mathrm{REC} \left(\%\right),$ was calculated from the following relationship:

3 $$\mathrm{REC} =\frac{{q}_{e,i}-{q}_{e,f}}{{q}_{e,i}}\times 100$$

where ${q}_{e,i}$ (mg/g) is the initial adsorption capacity of adsorbent, and ${q}_{e,f}$ (mg/g) is the final adsorption capacity, after the regeneration experiment had been completed. The PHE removal efficiency of the adsorbent, $\mathrm{RE} (\%)$, was calculated from the following relationship:

4 $$\mathrm{RE}=\left(\frac{{C}_{0}-{C}_{e}}{{C}_{0}}\right)\times 100$$

### Multi-compartment mass transfer model

To model the PHE sorption dynamics on the surface of the porous particles of adsorbent, four mass transfer processes are taken into account (Lesage et al. 2010): (i) the mass transfer from the bulk to the external surface of particles, which is governed by a film diffusion coefficient, ${k}_{f}$; (ii) the PHE sorption on the external surface of particles; (iii) the mass transfer inside the pore structure of particles through molecular diffusion in macro- /meso-pores, and surface diffusion in micro-pores, governed by the effective pore, ${D}_{e}$, and surface, ${D}_{s}$, diffusion coefficient, respectively; (iv) the PHE sorption on the pores. Assuming that the last step of sorption is a reversible linear and instantaneous process with equilibrium constant, ${K}_{e}$, the space- and time-dependent mass balances of dissolved and adsorbed PHE concentrations over the various compartments can be transformed into a system of particle-averaged ordinary differential equations (Goto et al. 1990; Lesage et al. 2010), accounting for the overall mass transfer coefficients, ${k}_{M}$ and ${k}_{m}$, for macro-/meso-pores and micro-pores, respectively. Specifically, the transient responses of the PHE concentration in bulk, ${C}_{b}$, mean PHE concentration in macro-pores, $\overline{{C }_{M}}$, mean PHE concentration adsorbed on meso-/macro-pores, $\overline{{Q }_{M}}$, mean PHE concentration adsorbed on micro-pores, $\overline{{Q }_{m}}$, and PHE concentration adsorbed on external grain surface, ${{Q }_{f}}$, are given by the following equations:

5 $$\frac{d{C}_{b}}{dt}={a}_{s}\left(\frac{\omega -1}{\omega }\right)\left[{k}_{M}\left({C}_{b}-\overline{{C }_{M}}\right)+{k}_{m}\left({C}_{b}-\frac{\overline{{Q }_{m}}}{{K}_{e}}\right)+{k}_{f}\left({C}_{b}-\frac{\overline{{Q }_{f}}}{{K}_{e}}\right)\right]$$

6 $$\frac{d\overline{{C }_{M}}}{dt}=\frac{{a}_{s}{k}_{M}}{{\varepsilon }_{M}+{S}_{M}{\rho }_{g}{K}_{e}}\left({C}_{b}-\overline{{C }_{M}}\right)$$

7 $$\frac{d\overline{{Q }_{M}}}{dt}=\frac{{a}_{s}{k}_{M}{K}_{e}}{{\varepsilon }_{M}+{S}_{M}{\rho }_{g}{K}_{e}}\left({C}_{b}-\overline{{C }_{M}}\right)$$

8 $$\frac{d\overline{{Q }_{m}}}{dt}=\frac{{a}_{s}{k}_{m}{K}_{e}}{{S}_{m}{\rho }_{g}}\left({C}_{b}-\overline{\frac{{Q }_{m}}{{K}_{e}}}\right)$$

9 $$\frac{d{Q}_{f}}{dt}=\frac{{a}_{s}{k}_{f}}{{S}_{f}{\rho }_{g}}\left({C}_{b}-\frac{{Q}_{f}}{{K}_{e}}\right)$$

To distinguish the separate contribution of each compartment to the total sorption of PHE, the following quantities are defined:

10a $${q}_{M}=\overline{{Q }_{M}}{S}_{M}$$

10b $${q}_{m}=\overline{{Q }_{m}}{S}_{m}$$

10c $${q}_{f}={Q}_{f}{S}_{f}$$

The total adsorbed mass, ${q}_{t}$, is obtained with a mass balance over the various compartments of the particle, including the dissolved PHE remaining in macro-pores, namely:

11 $${q}_{t}={q}_{M}+{q}_{m}+{q}_{f}+{V}_{M}\overline{{C }_{M}}$$

Having determined the total specific surface area, ${S}_{\mathrm{BET}}$, from N_2_ sorption, the specific surface area and pore volume for the macro-/meso-porosity, ${S}_{M}$ and ${V}_{M}$, respectively, can be approximated from the corresponding quantities provided from MIP data, ${S}_{\mathrm{MIP}}$ and ${V}_{\mathrm{MIP}}$, while the specific surface area for micro-pores can be estimated by:

12 $${S}_{m}={S}_{BET}-{S}_{M}$$

The external specific surface area,${a}_{s}$, of spherical particles with their radii, ${r}_{g}$, following a number-based distribution function, ${f}_{N}\left({r}_{g}\right)$, is given by:

13 $$a_s=\int_0^\infty f_N\left(r_g\right)\left(\frac3{r_g}\right)dr_g$$

and the external surface area of particles, ${S}_{f}$, is estimated approximately by the relationship:

14 $${S}_{f}=\frac{{a}_{s}}{{\rho }_{g}}$$

where ${\rho }_{g}$ is the bulk density of porous particles. The macro-/meso-porosity, ${\varepsilon }_{M}$, and the fraction of the bulk liquid volume, $\omega$, are defined by:

15 $${\varepsilon }_{M}={\rho }_{g}{V}_{M}$$

and

16 $$\omega =\frac{1}{1+{m}_{V}{\rho }_{g}}$$

respectively, where ${m}_{V}$ is the concentration of particles in the liquid phase.

The foregoing system of ordinary differential equations (ODEs), Eqs. (5)–(9), was solved numerically with finite differences by using the initial condition: ${C}_{b}={C}_{b0}$, $\overline{{C }_{M}}=\overline{{Q }_{M}}=\overline{{Q }_{m}}={{Q }_{f}}=0,$ at $t=0$. The calculated transient response of the concentration in the bulk, ${C}_{b}\left(t\right)$, was fitted to experimental results of kinetic sorption tests to estimate the parameter values: ${K}_{e},{k}_{M},{k}_{m},{k}_{f}$. The corresponding values of effective pore, ${D}_{e}$, and surface, ${D}_{s}$, diffusion coefficients were obtained with the relationships (Lesage et al. 2010):

17 $${D}_{e}=\frac{{r}_{P}}{5\left(\frac{1}{{k}_{M}}-\frac{1}{{k}_{f}}\right)}$$

18 $${D}_{s}=\frac{{r}_{P}}{5\left(\frac{1}{{k}_{m}}-\frac{1}{{k}_{f}}\right)}$$

## Results and discussion

### Tests on the adsorption capacity of the adsorbents

The mass loss of initial raw material that was converted to AC is indicative of the percentage of solid material that was replaced by porosity, and is of key importance when examining the cost-effectiveness and sustainability of adsorbent production compared to its use for environmental restoration. In this framework, the mass loss of the activated carbon prepared from CW, during the two stages of treatment (pyrolysis and washing until reaching at a neutral pH), was estimated from weights (Table 1). It was observed that after washing, the mass losses increased by ~ 30% compared to the mass losses after pyrolysis, especially for the adsorbent treated with NaOH (CWAC-NaOH-800).

**Table 1:** Mass loss of CW-based adsorbents after treatment stages

Adsorbent	Mass loss after pyrolysis (%)	Mass loss after washing (%)
CWAC-800	80.0	-
CWAC-NaOH-800	58.2	83.2
CWAC-H_3_PO_4_-800	64.2	68.4

**Fig. 3:**
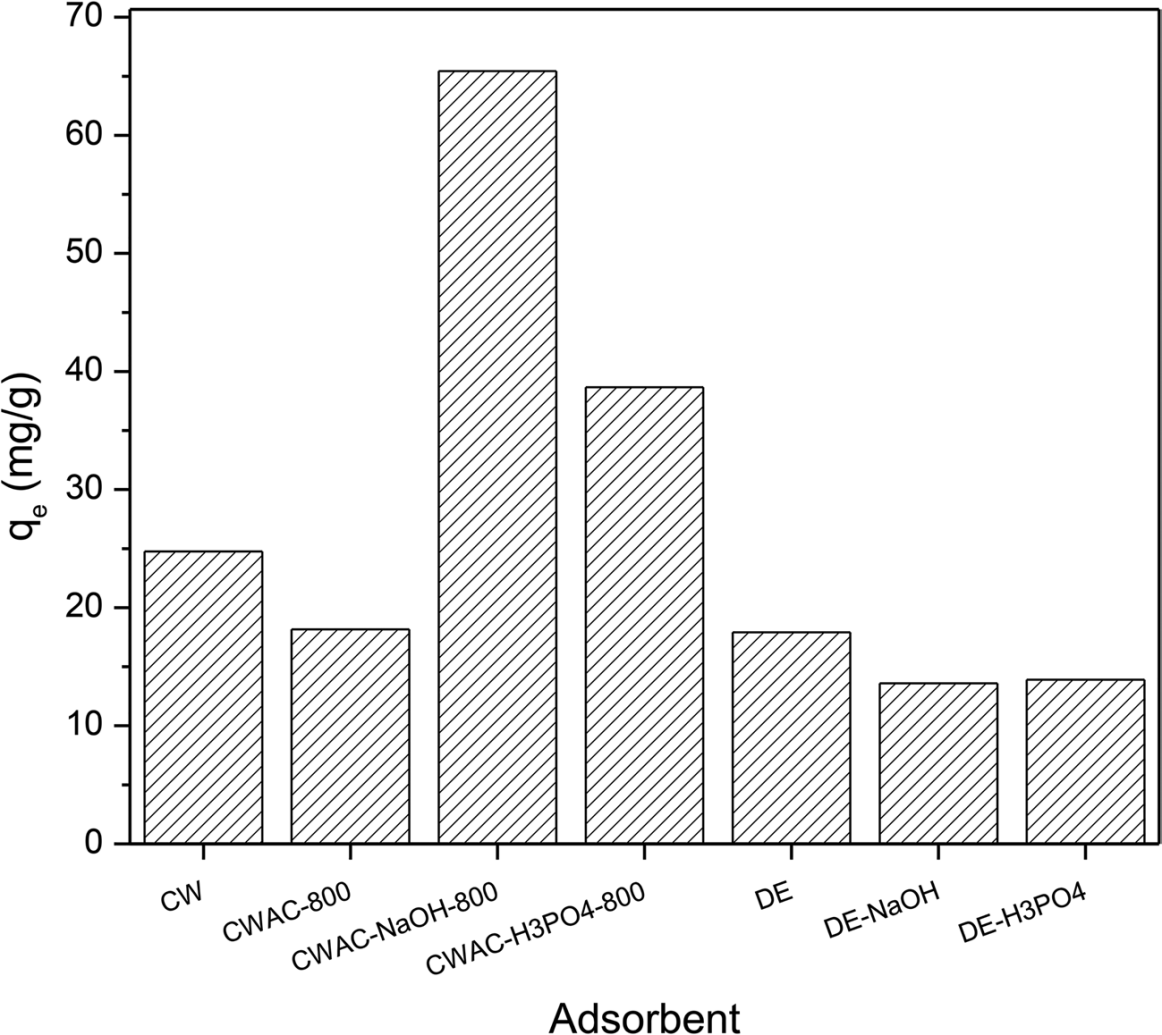
Adsorption capacity of untreated CW and physically and/or chemically treated CW (CW, untreated coffee waste; CWAC-H_3_PO_4_-800, CW treated with H_3_PO_4_ and pyrolyzed at 800 °C; CWAC-NaOH-800, CW treated with NaOH and pyrolyzed at 800 °C; CWAC-800, CW pyrolyzed at 800 °C) and untreated DE and chemically treated DE (DE-NaOH, DE treated with NaOH; DE-H_3_PO_4_, DE treated with H_3_PO_4_) (*C*_0_ = 30 mg/L, adsorbent dosage = 0.5 g/L, treatment time = 180 min, pH = 5.6)

Initially, sorption tests were performed to compare the PHE sorption capacity of all adsorbents, i.e., CW, CWAC-800, CWAC-NaOH-800, CWAC-H_3_PO_4_-800, DE, DE-NaOH, DE-H_3_PO_4_. Based on the measured PHE adsorption capacity, the most efficient adsorbent was selected and used furthermore. The initial concentration of PHE was 30 mg/L, the adsorbent dosage was 0.5 g/L, the initial pH of the PHE solution was 5.6, and the equilibrium contact time was set to 180 min. It was observed that the highest adsorption capacity was achieved with the use of CW treated with NaOH and pyrolyzed at 800 °C (CWAC-NaOH-800) as shown in Fig. 3. Then, this adsorbent was used for kinetic and equilibrium adsorption and regeneration studies. The mass-based grain size distribution of CWAC-NaOH-800 was measured over the size range 50–1000 μm with a series of sieves (Table 2). The measured TOC value in water equilibrated with CWAC-NaOH-800 was found equal to 0.4 mg/L. Although there are no specific permissible limits of TOC in treated water, and accounting for the intense mixing of grains with water, this value is considered quite low, indicating that no additional hazardous components were released in water from CWAC-NaOH-800. It is also worth mentioning that any water-soluble compounds that might remain inside the pores of CWAC-NaOH-800 after the pyrolysis were withdrawn with washing through vacuum filtration (Fig. 1a).

**Table 2:** Granulometry of CWAC-NaOH-800

Size range (μm)	Mass percentage (%)
50–125	5.08
125–250	26.13
250–500	46.04
500–1000	23.46

### Physicochemical and morphological characterization of the adsorbents

#### N_2_ sorption isotherms and mercury intrusion porosimetry

**Fig. 4:**
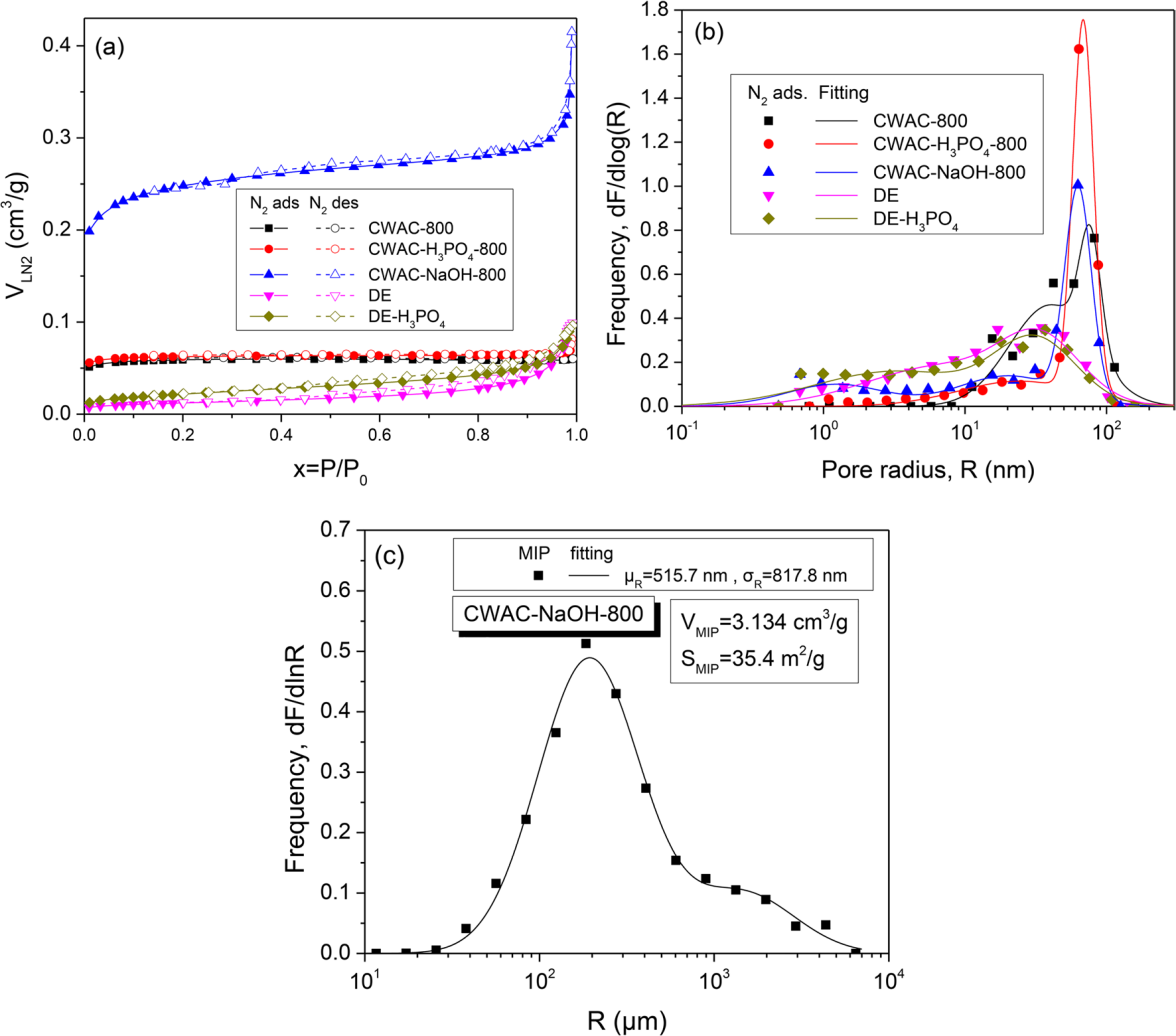
**a** N_2_ sorption isotherms. **b** Pore size distributions obtained from N_2_ adsorption curve. **c** Pore size distribution obtained from Hg intrusion porosimetry

The N_2_ adsorption–desorption isotherms along with the pore radius distribution, obtained from the adsorption branch, are shown in Fig. 4 a and b, while the specific surface area, total pore volume, and statistical moments of the size distributions are given in Table 3. It is worth mentioning that all pore size distributions were fitted with composite bimodal (CWAC-800, CWAC-H_3_PO_4_-800) or trimodal (CWAC-NaOH-800, DE, DE-H_3_PO_4_) distribution functions, composed of two or three log-normal component distribution functions (Fig. 4b; Table 3). No meso-porosity (2 nm < *R* < 50 nm) and micro-porosity (*R* < 2 nm) or respectable specific surface area (Table 3) is evident for two materials (CW, DE-NaOH). In addition, the meso- and micro-porosity of most materials is limited, except of CWAC-NaOH-800 (Fig. 4a; Table 3), which exhibited the highest pore volume, *V*_LN2_, the highest specific surface area (Fig. 4a; Table 3), and the highest sorption capacity for PHE (Fig. 3). The complete pore size distribution and pore volume of CWAC-NaOH-800, including the full macro-pore region, were obtained with differentiation of the high-pressure mercury intrusion curve and are shown in Fig. 4c. The fitting curve is a composite bimodal distribution composed of two log-normal component distribution functions. Evidently, the pore volume detected by N_2_ sorption is much less than that detected by MIP (Fig. 4a, c), which in practice coincides with the total pore volume of meso-/macro-porosity. The high pore volume of CWAC-NaOH-800 (Fig. 4c) is consistent with the large mass loss during its fabrication (Table 1).

**Table 3:** Pore space properties of the adsorbents

Adsorbent	${S}_{\mathrm{BET}}$ (m^2^/g)	*V*_P, LN2_ (cm^3^/g)	Mean radius *μ*_*R*_ (nm)	Standard deviation *σ*_*R*_ (nm)
CW	0.25	0.0024	-	-
CWAC-800	159.0	0.0604	56.70	35.65
CWAC-H_3_PO_4_-800	167.5	0.0815	61.0	32.70
CWAC-NaOH-800	676.5	0.415	43.10	35.35
DE	34.9	0.0991	25.84	32.95
DE-H_3_PO_4_	63.5	0.0964	22.03	25.12
DE-NaOH	0.9	0.0064	-	-

#### Scanning electron microscopy images of the adsorbents

**Fig. 5:**
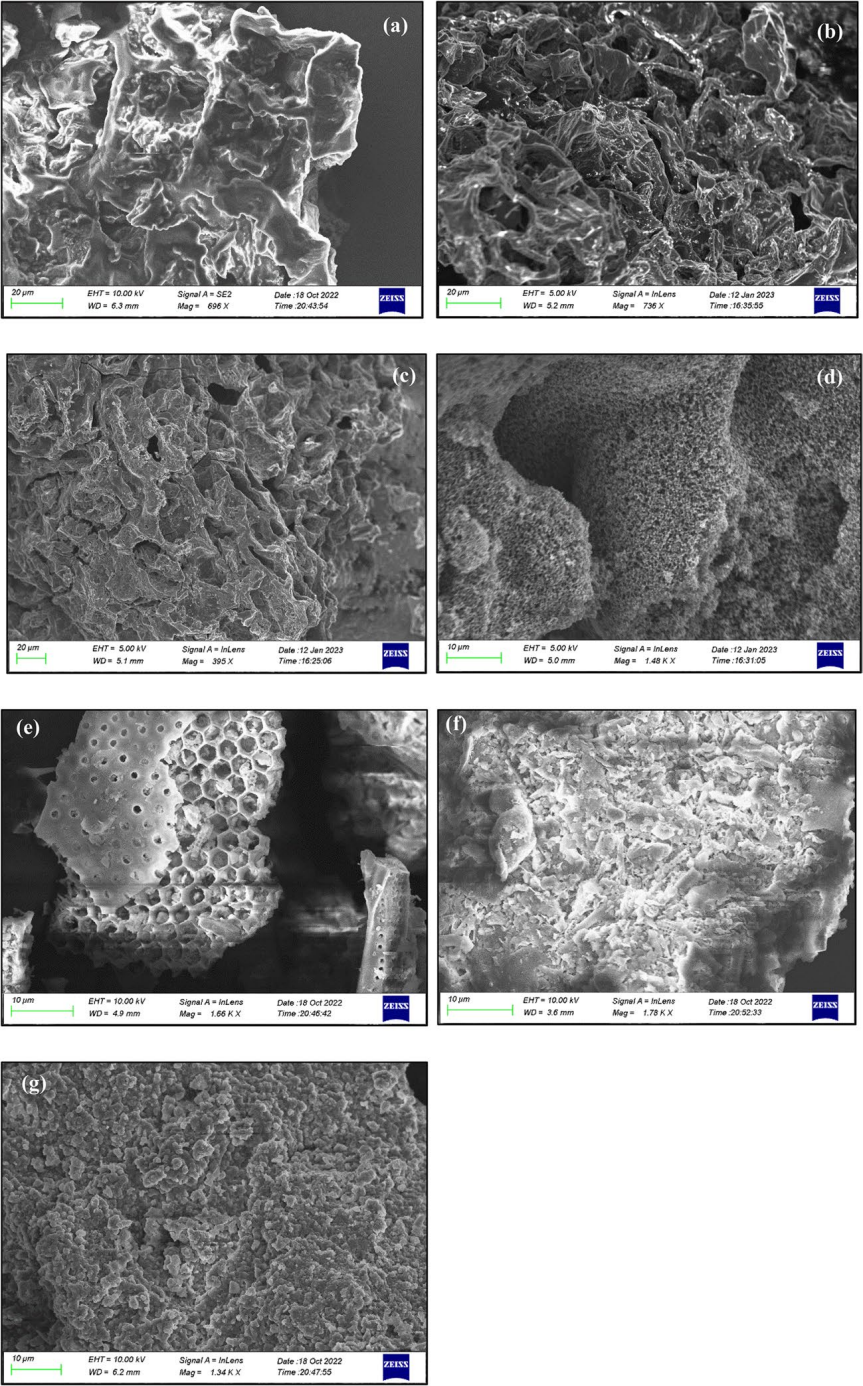
SEM images of the adsorbents: **a** CW, **b** CWAC-800, **c** CWAC-H_3_PO_4_-800, **d** CWAC-NaOH-800, **e** DE, **f** DE-H_3_PO_4_, **g** DE-NaOH

SEM is one of the most important characterization techniques providing information for the morphology of the materials, which is crucial for the determination of the interaction mechanism of adsorbate molecules and adsorbent. The SEM images of the adsorbents are presented in Fig. 5. The surface of CW (Fig. 5a) is non-porous and rough, which is also confirmed from the very low ${S}_{\mathrm{BET}}$ value (Table 3). When CW is pyrolyzed, without pre-treatment or pre-treating with H_3_PO_4_, a macro-porous network is developed (Fig. 5b, c) and a higher ${S}_{\mathrm{BET}}$ value is measured (Table 3). When CW is pretreated with NaOH and then pyrolyzed, a dense network of macro- and meso-pores (Fig. 5d), of high specific surface area (Table 3) and total pore volume (Fig. 4c), is created. In adsorbents generated from DE, a very different surface morphology is evident compared to those observed in the CW-based ones (Fig. 5e–g). DE has cellular-shaped macro-pores blocked by impurities (Fig. 5e). After its treatment with H_3_PO_4_, the macro-porous structure becomes disordered with the creation of agglomerates (Fig. 5f) (Zhao et al. 2020) that enhance the contribution fraction of meso-pores to total porosity (Fig. 4b), and increase weakly the ${S}_{\mathrm{BET}}$ value (Table 3). After the treatment of DE with NaOH, the pore structure is destructed (Fig. 5g) and this is reflected in the very low ${S}_{\mathrm{BET}}$ and ${V}_{\mathrm{LN}2}$ values (Table 3). The changes observed in the morphology of the adsorbents are fully consistent with the pore space characteristics probed by N_2_ isotherms (Table 3; Fig. 4a, b) and MIP data (Fig. 4c). On the other hand, with the specific surface area and pore volume increasing, the PHE sorption capacity of materials has the tendency to increase (Fig. 3). Obviously, a well-developed pore network, like that generated during the synthesis of CWAC-NaOH-800, is of key importance for the enhancement of the adsorption of PHE.

#### Attenuated total reflectance-Fourier transform infrared and Raman spectra of the adsorbents

**Fig. 6:**
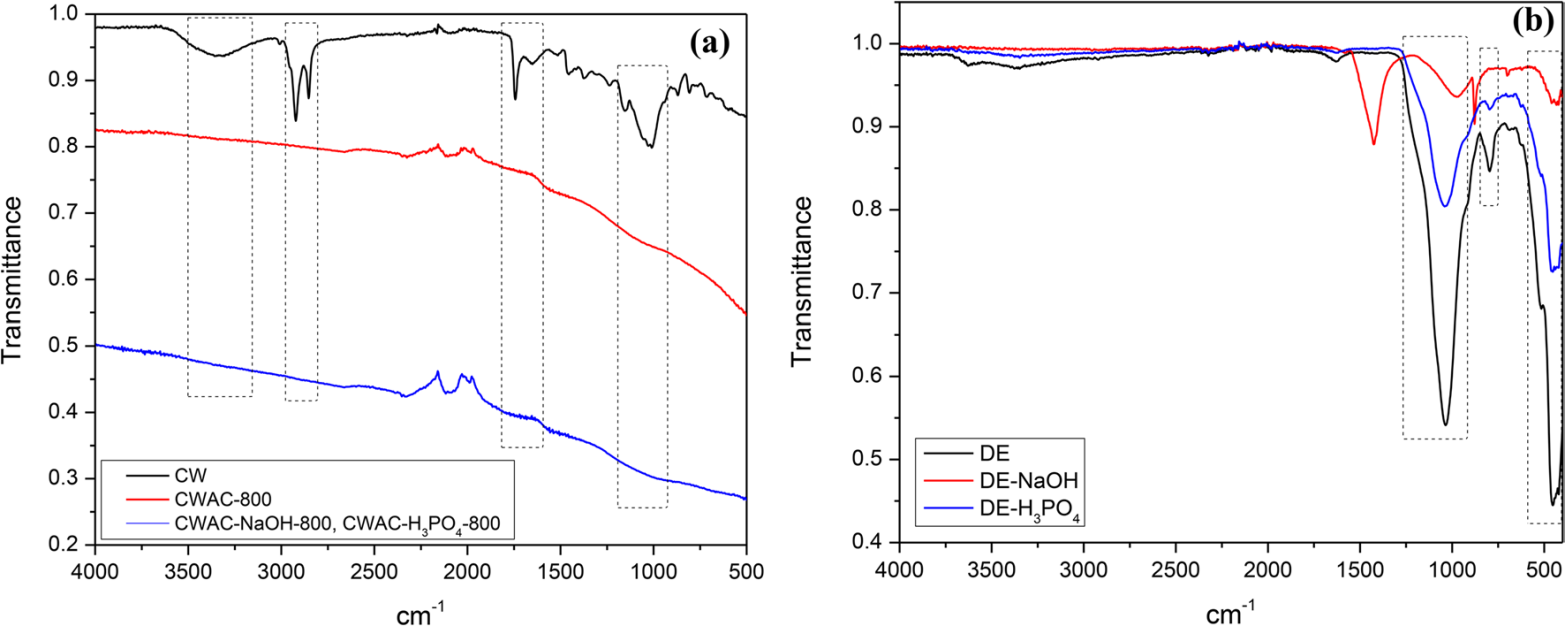
ATR-FTIR spectra of the adsorbents: **a** CW, CWAC-800, CWAC-NaOH-800, and CWAC-H_3_PO_4_-800; and **b** DE, DE-NaOH, and DE-H_3_PO_4_

The ATR-FTIR spectra of the adsorbents are shown in Fig. 6. The spectra of CW and CWACs (CW, CWAC-800, CWAC-NaOH-800, CWAC-H_3_PO_4_-800) are presented in Fig. 6a. Regarding CW, the bands at around 3600–3070 cm^−1^ (peak at 3330 cm^−1^) and 3000–2800 cm^−1^ (peaks at 2920 and 2850 cm^−1^) are characteristic of lignocellulosic materials. More specifically, the first band corresponds to O–H stretching vibration of the hydroxyl group and the second is associated with symmetrical and asymmetrical C-H stretching of methoxyl groups. The peak at 1740 cm^−1^ shows the C = O stretching of the carbonyl groups and the peak at 1020 cm^−1^ corresponds to C-O vibration of alcohol. At the spectra of the CWACs, all characteristic peaks were eliminated, indicating the transformation of CW into carbon produced at the high pyrolysis temperature of 800 °C (Chwastowski et al. 2020; Rosson et al. 2021).

**Fig. 7:**
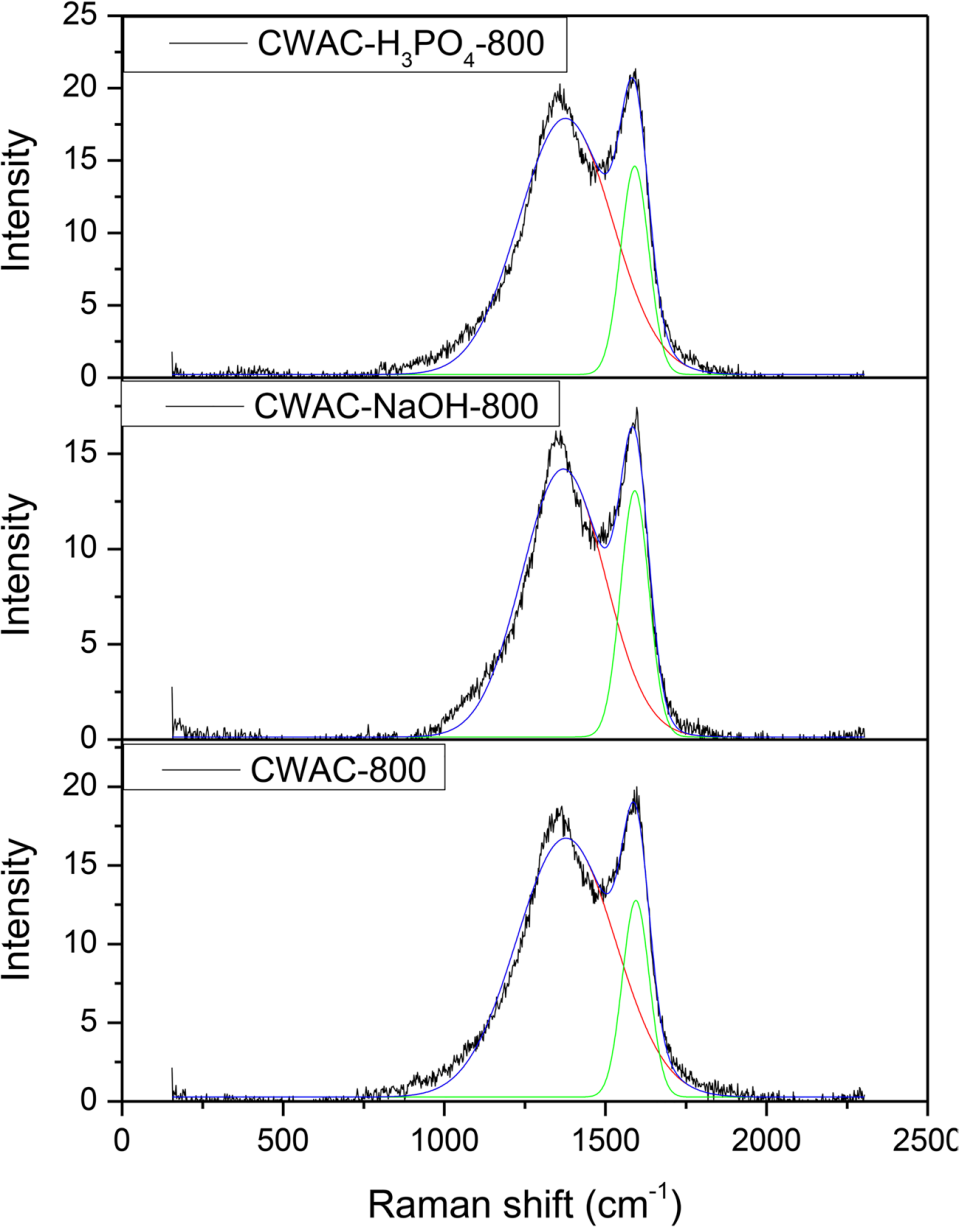
Raman spectra of the three ACs produced from CW: CW, CWAC-800, CWAC-NaOH-800, CWAC-H_3_PO_4_-800

To understand more deeply the carbonaceous nature of the adsorbents, the Raman spectra of the three activated carbons, CWAC-800, CWAC-NaOH-800, and CWAC-H_3_PO_4_-800, were recorded (Fig. 7). The degree of graphitization was determined based on the excitement amount of the two characteristic bands which are present at all Raman spectra, the D- and G-band. The ratio ${I}_{D}/{I}_{G}$ is indicative of the feature defects of AC (Mazrouaa et al. 2019) and reveals the degree of disorder and graphitization (Rosson et al. 2021; Alcaraz et al. 2021; Zięzio et al. 2020). The band centered at 1350 cm^−1^ is the D-band which is derived from the defects and is related to the amorphous carbon structure, while the band centered at 1588 cm^−1^ is the G-band which indicates the degree of disorder of the material. From the analysis of the spectra through deconvolution of the peaks, useful structural information about the adsorbents was obtained. The deconvolution was carried out with the fitting of the Gaussian distribution to the spectra (${R}^{2}$=0.989) (Fig. 7), and the calculation of the peak intensity ratio ${I}_{D}/{I}_{G}$ for the three CWACs. The ratio ${I}_{D}/{I}_{G}$ was equal to 1.32 for CWAC-800, 1.08 for CWAC-NaOH-800, and 1.23 for CWAC-H_3_PO_4_-800. The CWAC-NaOH-800, with the smallest intensity ratio, is expected to have the fewest defects.

The ATR-FTIR spectra of DE and DE-H_3_PO_4_ are very similar to each other, differing only with respect to the intensities of peaks (Fig. 6b), while the NaOH treatment of DE led to the shift of some of the characteristic peaks towards smaller wavenumbers, showing much lower intensities from the other two adsorbents (Fig. 6b). The peak at 460 cm^−1^, which is common for the three adsorbents, corresponds to bending vibration of Si–O-Si bonds. The peaks at around 794 cm^−1^ and 1040 cm^−1^ are attributed to Si–O symmetric stretching vibration and Si–O in plane stretching vibration, respectively. The very low intensity peak at 1645 cm^−1^, which appears only at the spectrum of untreated DE, corresponds to OH stretching and bending vibrations of molecular water (Zhao et al. 2020; Dinh Du and Danh 2021; Zhao et al. 2015).

### Adsorption of PHE onto CWAC-NaOH-800

#### Effect of the initial pH and contact time on the adsorption capacity of CWAC-NaOH-800

**Fig. 8:**
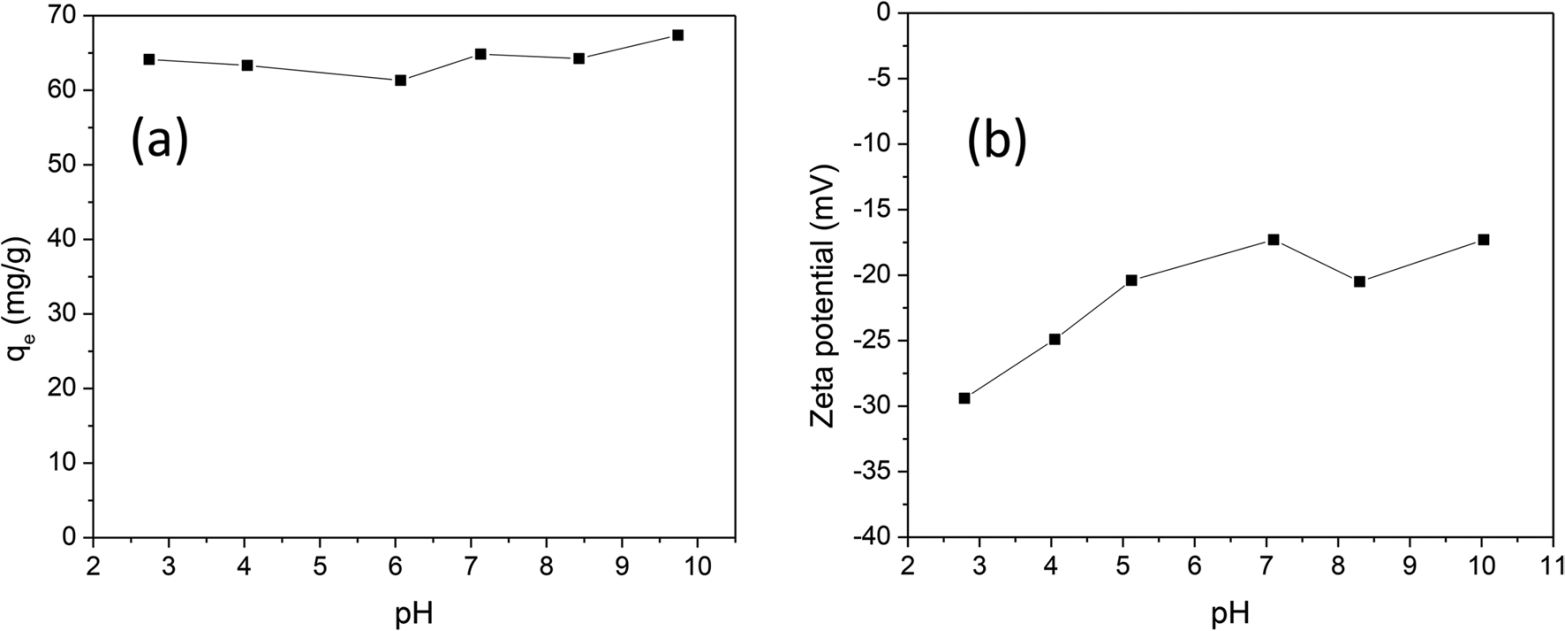
**a** Effect of pH on PHE adsorption onto CWAC-NaOH-800 (*C*_0_ = 30 mg/L, contact time = 3 h, adsorbent dosage = 0.5 g/L). **b** Variation of the *ζ*-potential of CWAC-NaOH-800 as a function of pH

**Fig. 9:**
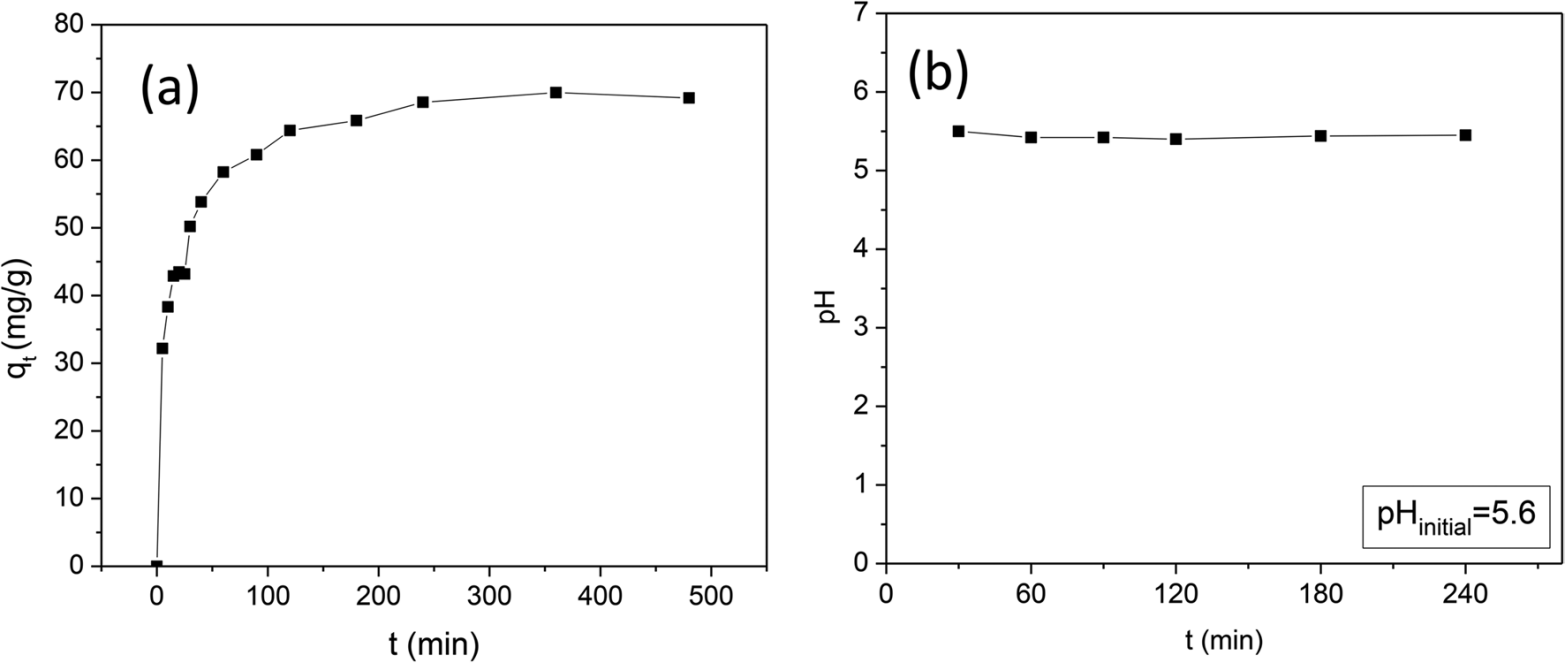
**a** Effect of contact time on PHE adsorption onto CWAC-NaOH-800 (*C*_0_ = 30 mg/L, pH = 5.6, adsorbent dosage = 0.5 g/L). **b** Variation of pH during the adsorption of PHE onto CWAC-NaOH-800 (*C*_0_ = 30 mg/L, adsorbent dosage = 0.5 g/L)

The effect of the pH of the PHE solution on the adsorption capacity of CWAC-NaOH-800, ${q}_{e}$ (mg/g), at equilibrium, is shown in Fig. 8. No remarkable variation of the ${q}_{e}$ value was observed with the pH increasing from 2 to 10 (Fig. 8a). The lack of polarity and ionization of PHE molecules reduces the possibility of strong electrostatic interactions between them and the charged surface of the adsorbent. This result has been confirmed by other researchers, as well (Esfandiar et al. 2021; Lamichhane et al. 2016; Guo et al. 2017; Shi et al. 2013)*.* By measuring the *ζ*-potential of CWAC-NaOH-800, it was confirmed that the surface charge of the adsorbent was insensitive to the pH variation over the range 2–10 (Fig. 8b). In addition, no isoelectric point was identified, revealing the lack of any preference of PHE molecules for sorption under acidic or alkali conditions. Likewise, Shi et al. (2013) highlighted that the absence of polarity of naphthalene molecules resulted in dispersive interactions, related to the polyaromatic structure of the PAH and the graphene layer of the AC. Moreover, the pH was monitored during the adsorption of PHE and no sensible change was observed during a period of 4 h (Fig. 9b), confirming that pH does not affect the PHE sorption. In this manner, all next kinetic and equilibrium sorption experiments were conducted at initial pH equal to 5.6, which was the initial pH value of PHE solution.

The time needed for the system of adsorbate-adsorbent to reach equilibrium was determined with kinetic experiments by shaking the PHE solution for 8 h and keeping the initial PHE concentration constant at 30 mg/L with the pH equal to 5.6. As shown in Fig. 9a, the amount of PHE adsorbed on CWAC-NaOH-800 was increased rapidly within the first 60 min, probably due to the abundance of available adsorption sites on the surface of CWAC-NaOH-800 (Hasan et al. 2023b). Then, the adsorption capacity was increased slowly, most likely because of pore diffusion and decrease of surface sites for sorption; it reached a plateau as the adsorption sites were saturated (Hasan et al. 2023a), so that finally equilibrium was established after 3 h, which was the duration of all experiments. A detailed analysis of the dynamics of sorption mechanisms is described in the section “Simulation of sorption dynamics and parameter estimation” by placing emphasis on the quantitative interpretation of the diffusion steps of the whole process.

#### Effect of the initial PHE concentration on the adsorption capacity of CWAC-NaOH-800

**Fig. 10:**
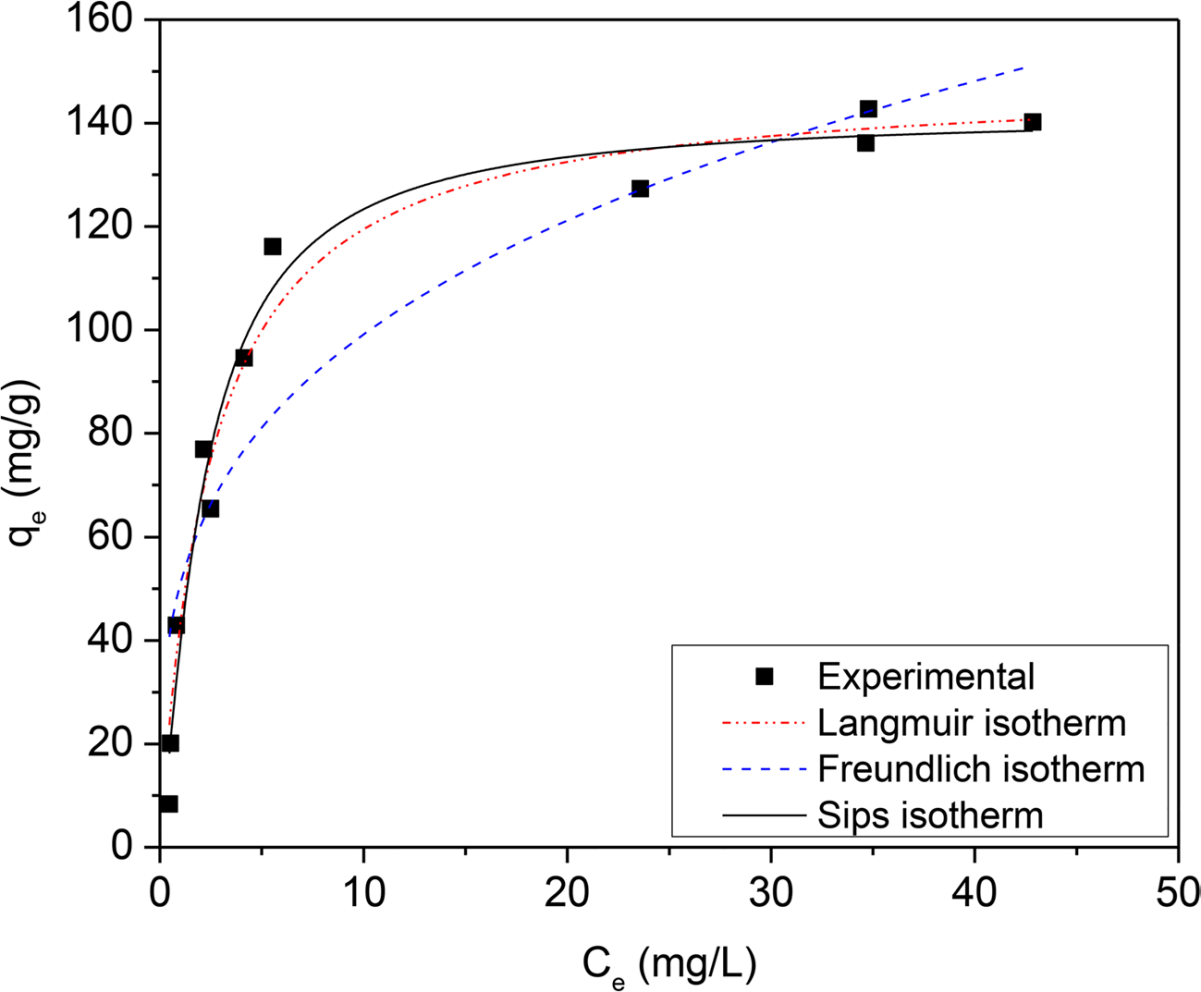
Effect of PHE initial concentration on its adsorption onto CWAC-NaOH-800 (contact time = 3 h, pH = 5.6, adsorbent dosage = 0.5 g/L) and isotherm fitting with various models

The performance of CWAC-NaOH-800 was evaluated by varying the initial concentration of PHE from 5 to 110 mg/L at initial pH = 5.6, for an equilibrium contact time of 3 h. As shown in Fig. 10, the adsorption capacity increases with the PHE concentration increasing, thanks to the increasing driving force (concentration difference) that overbalances the mass transfer resistance between the bulk liquid phase and the solid matrix. The interactions between the adsorbate molecules and adsorbent were elucidated by fitting the equilibrium sorption data to the non-linear form of Langmuir (Langmuir 1918), Freundlich (Freundlich 2017), and Sips (Tzabar and ter Brake 2016) isotherms, which are described by the following equations:

Langmuir isotherm

19 $${q}_{e}=\frac{{q}_{\mathrm{max}}{K}_{L}{C}_{e}}{1+{K}_{L}{C}_{e}}$$

where ${q}_{\mathrm{max}}$ (mg/g) is the maximum adsorption capacity of the adsorbent for monolayer coverage and ${K}_{L}$ (L/mg) is the Langmuir adsorption constant related to the affinity of binding sites and the free energy of adsorption. The Langmuir isotherm describes monolayer adsorption on structurally homogenous materials where no interaction between adsorbed molecules occurs. Moreover, it assumes that the adsorption sites are energetically identical and intermolecular forces decrease rapidly with the distance from the adsorption surface (Awual and Yaita 2013; Foo and Hameed 2012).

Freundlich isotherm

20 $${q}_{e}={K}_{F}{C}_{e}^{{~}^{1}\!\left/ \!{~}_{n}\right.}$$

where ${K}_{F}$ ((mg/g)(L/mg)^1/*n*^) is the Freundlich adsorption constant related to the maximum adsorption capacity of the adsorbent and ${~}^{1}\!\left/ \!{~}_{n}\right.$ is a constant related to the intensity of adsorption and varying with the heterogeneity of its surface. The Freundlich isotherm describes multilayer adsorption where interactions between adsorbed molecules take place and the distribution of adsorption heat and affinities over the heterogeneous surface are non-uniform (Aggelopoulos et al. 2017).

Sips isotherm

21 $${q}_{e}=\frac{{q}_{\mathrm{max}}{K}_{s}{C}_{e}^{{~}^{1}\!\left/ \!{~}_{n}\right.}}{1+{K}_{s}{C}_{e}^{{~}^{1}\!\left/ \!{~}_{n}\right.}}$$

where ${K}_{S}$ (L/mg)^1/*n*^ is the Sips adsorption constant and *n* is a dimensionless parameter that characterizes the heterogeneity of the adsorbate-adsorbent system, when 0 < $n$<1. When $n$ = 1, the Sips equation reduces to the Langmuir equation, indicating a homogeneous adsorption process (Kumara et al. 2014). The Sips isotherm is a hybrid isotherm of Langmuir and Freundlich isotherms and describes localized adsorption without interactions between adsorbate and adsorbent (Tzabar and ter Brake 2016).

Data of PHE sorption onto CWAC-NaOH-800 at equilibrium were fitted with Langmuir, Freundlich, and Sips models (Fig. 10) and the estimated parameter values are shown in Table 4. The best fit to the experimental data was achieved with the Sips isotherm (${R}^{2}$ = 0.971) indicating that the adsorbate-adsorbent system is heterogenous with heterogeneity exponent, $n$, equal to 0.8 and maximum adsorption capacity, ${q}_{\mathrm{max}}$ (mg/g), equal to 141.94 mg/g.

**Table 4:** Isotherm parameters for the adsorption of PHE onto CWAC-NaOH-800

Langmuir			Freundlich			Sips			
${q}_{\mathrm{max}}$ mg/g	${K}_{L}$ L/mg	${R}^{2}$	${K}_{F}$ (mg/g)(L/mg)^1/*n*^	$1/n$	${R}^{2}$	${q}_{\mathrm{max}}$ mg/g	${K}_{s}$ (L/mg)^1/*n*^	$n$	${R}^{2}$
148.70	0.409	0.968	50.87	0.289	0.849	141.94	0.381	0.806	0.971

In Table 5, the maximum PHE adsorption capacity of CWAC-NaOH-800 (present work) is compared to corresponding ones mentioned earlier for the removal of PHE from aqueous solutions. The adsorption capacity of CWAC-NaOH-800 is comparable to or even higher than those achieved with other adsorbents, showing its suitability as an adsorbent for the removal of PHE from water streams.

**Table 5:** Comparative analysis of the PHE adsorption capacity of the present study with regard to earlier ones

Adsorbent	Adsorbent dosage (g/L)	Initial concentration (mg/L)	pH	Contact time (h)	${q}_{\mathrm{max}}$(mg/g)	Reference
AC from coffee waste (CWAC-NaOH-800)	0.5	5–110	5.6	3	141.9	This study
Biochar from excess sludge of sewage plant	0.2	0.1–10	7.0	2	87	(Guo et al. 2017)
Biochar from plant *Phragmites australis*	0.33	0.2–2	-	2	1.97	(Wang et al. 2020)
Magnetic biochar from rice husk	0.2	5–70	7	1	97.6	(Guo et al. 2018)
AC from petroleum coke	0.5	0.00025–0.005	-	18	0.046	(Wang et al. 2020)
AC from walnut shell	0.5–10	10	3	0.7	145.6	(Wu et al. 2020)
Stevensite, sepiolite	0.3 g	0.3–3.6	6	2	26.9, 0.1	(González-Santamaría et al. 2017)
Inorgano–organo-bentonite	1	0–350	6.5	4	${K}_{d}$= 355.3 L/g	(Ma and Zhu 2006)

#### Adsorbent regeneration

**Fig. 11:**
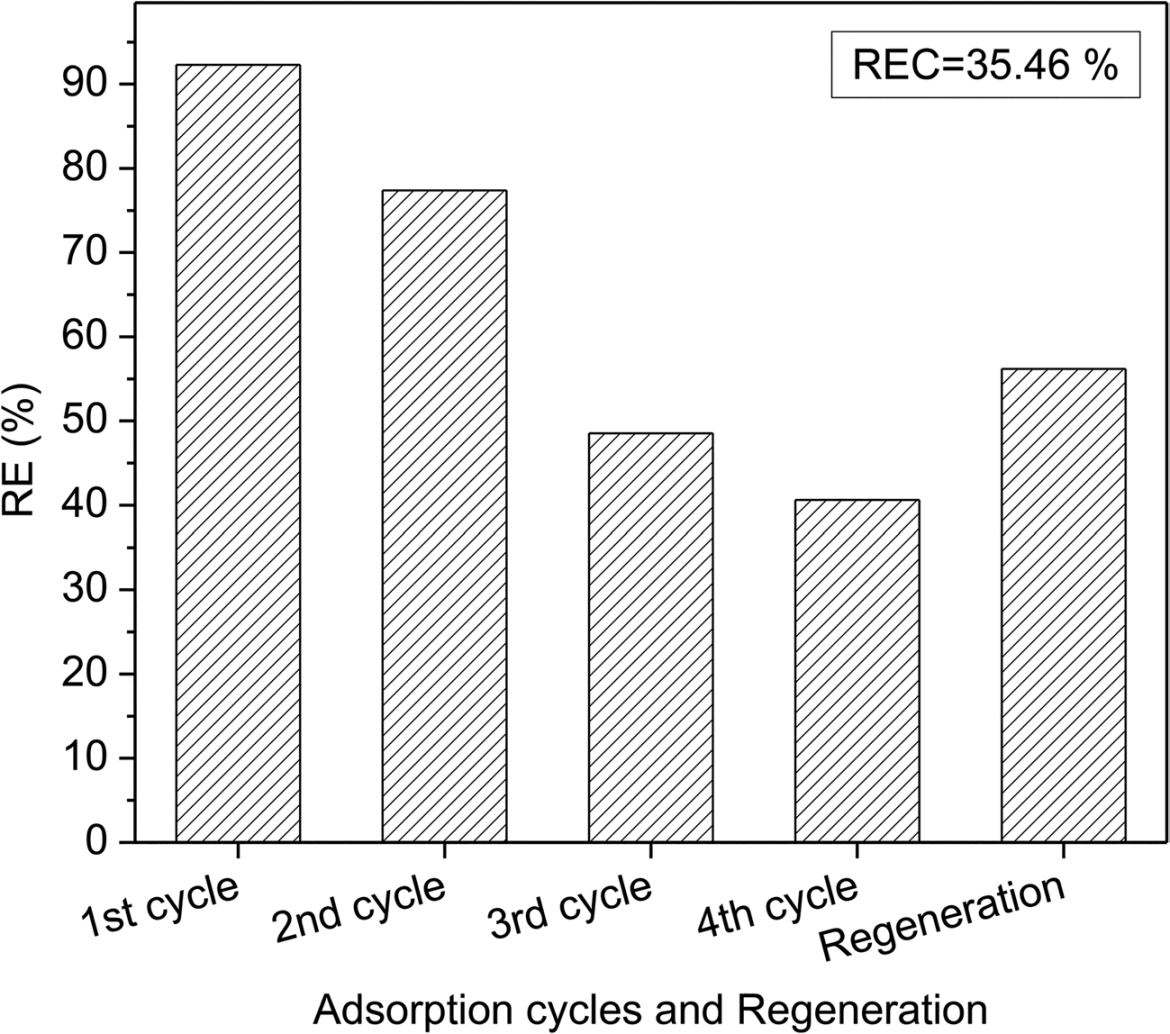
Adsorption capacity of CWAC-NaOH-800 and PHE removal efficiency during four adsorption cycles (*C*_0_ = 60 mg/L, contact time = 3 h, adsorbent dosage = 0.5 g/L, pH = 5.6) and CAP-assisted regeneration

The variation of the removal efficiency, $\mathrm{RE} (\%)$, during four adsorption cycles and after regeneration of the adsorbent CWAC-NaOH-800 is shown in Fig. 11. It can be observed that from the 1st to the 4th adsorption cycle the PHE removal efficiency decreased from 92.3 to 40.6%, while the adsorption capacity decreased from 108.11 to 47.50 mg/g. After three cycles of reuse, the adsorbent had not fully been saturated, and exhibited a satisfactory adsorption capacity even after the 4th cycle of adsorption. After the CAP-assisted regeneration test, the $\mathrm{RE}$ was enhanced by ~ 16% and the adsorption capacity was enhanced by ~ 22 mg/g, exceeding the corresponding value of the 3rd adsorption cycle (Fig. 11). The recovery of sorption capacity, $\mathrm{REC}$, was calculated and found equal to 35.5%, which is less than the ideal condition of a fully regenerated adsorbent, but still is considered a respectable value for the PHE-saturated CWAC-NaOH-800. The CAP-assisted adsorbent regeneration is attributed to the high oxidation potential of the plasma-generated reactive oxygen and nitrogen species (RONS) such as singlet oxygen (^1^O_2_), hydroxyl radical (**·**OH), oxygen (O), superoxide (**·**O_2_^−^), ozone (O_3_), nitrite (NO_2_^–^), nitrate (NO_3_^–^), peroxynitrite (ONOO^–^), and hydrogen peroxide (H_2_O_2_) (Giannoulia et al. 2023), leading to the re-creation of adsorption sites across the surface of adsorbent.

#### Simulation of sorption dynamics and parameter estimation

For the sake of computations, it is necessary to estimate a mean particle size for the granular CWAC-NaOH-800. The mass-based grain size distribution (Table 2) was converted into number-based grain size distribution which was used to calculate the mean value, standard deviation, and external surface area, ${a}_{s}$, Eq. (13) (Table 6). Kinetic sorption tests were carried out for three initial PHE concentrations (67, 82, and 112 mg/L). All parameter values resulting from experimental measurements and used in the model are shown in Table 6.

**Table 6:** Input data used in the multi-compartment model

$2{r}_{g} (\mathrm{\mu m})$	87.5	187.5	375	750	$\langle {r}_{g}\rangle =71 \mathrm{\mu m}$		
${f}_{N}$	0.6079	0.3178	0.0700	0.0043	${\sigma }_{g}$=44.3 μm		
${a}_{s}$(m^−1^)	${\rho }_{g}$(kg/m^3^)	${m}_{V}$(kg/m^3^)	${V}_{M}$(m^3^/kg)	${S}_{M}$(m^2^/kg)	${S}_{m}$(m^2^/kg)	${S}_{f}$(m^2^/kg)	${C}_{b0}$(kg/m^3^)
53,007	260.0	0.5	3.134 × 10^−3^	35.4 × 10^3^	641.1 × 10^3^	203.87	0.06705 0.08292 0.11245

**Fig. 12:**
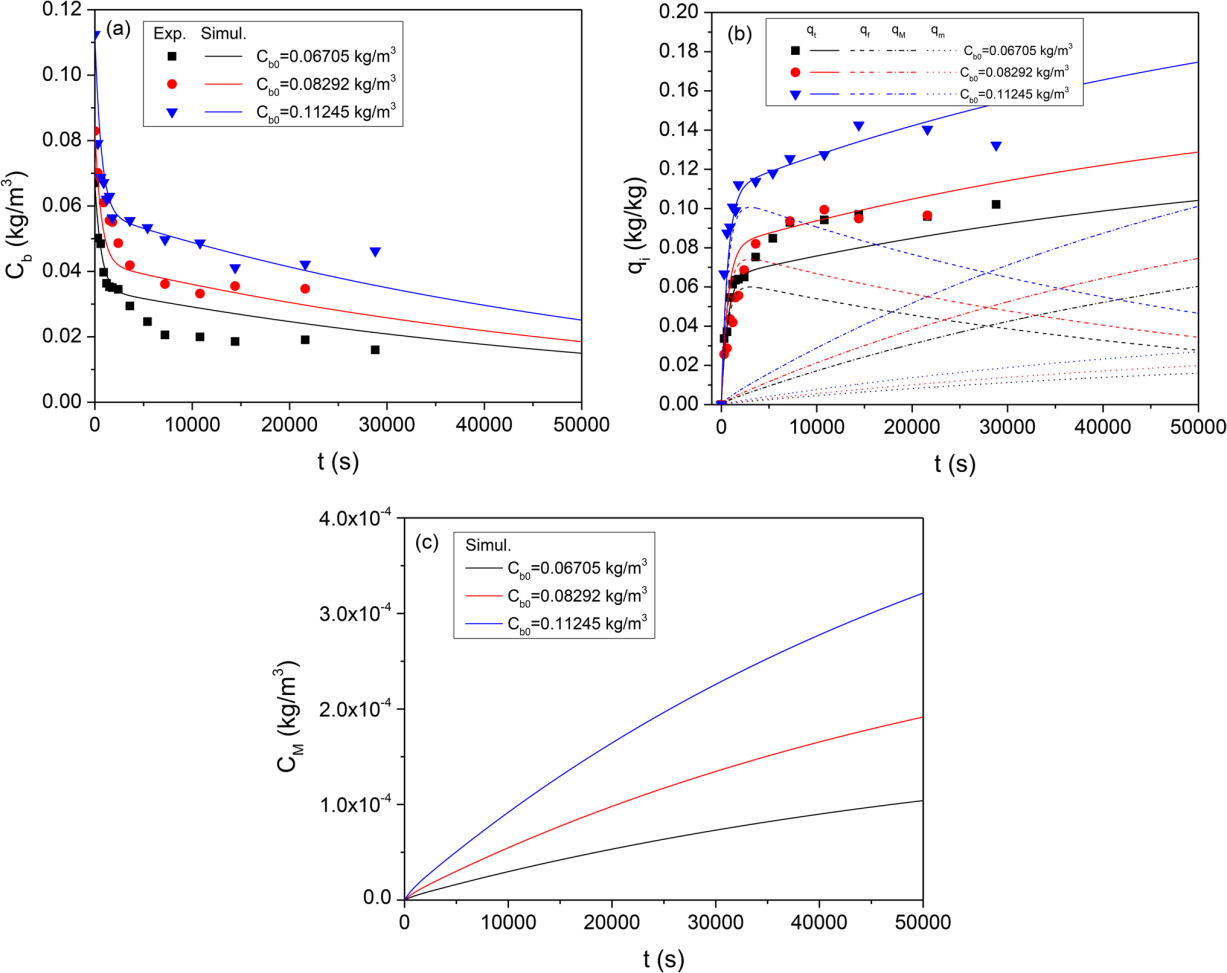
Comparison of experimentally measurements with predictions of multi-compartment model. **a** Transient response of PHE concentration in bulk liquid. **b** Transient response of the sorption capacity of PHE onto each compartment of CWAC-NaOH-800. **c** Transient response of the mean PHE concentration in meso -/macro-pores

Inverse modeling was done simultaneously for the three transient responses of the PHE concentration, measured for three initial concentrations, by solving the ODEs, Eqs. (5)–(9), with central finite differences and using the Bayesian estimator of Athena Visual Studio 14 to fit the numerical solution to experimental datasets (Stewart and Caracotsios 2008). The estimated values of the linear equilibrium constant for adsorption, ${K}_{e}$, and mass transfer coefficients ${k}_{M},{k}_{m}, \mathrm{} {k}_{f}$ are shown in Table 7 along with the effective diffusion coefficients calculated with Eqs. (17) and (18). The effective pore diffusion coefficient, ${D}_{e}$, is two orders of magnitude less than the corresponding molecular diffusion coefficient of PHE in water (${D}_{m}$=4.37 × 10^−10^ m^2^/s; Gustafson and Dickhut 1994). The simulated transient responses are compared with experimental results of kinetic tests in Fig. 12a, b. The independent contribution of each compartment (external surface, meso-/macro-porous region, micro-porous region) to the total sorption is shown in Fig. 12b.

**Table 7:** Estimated mass transfer coefficients

Parameter	CWAC-NaOH-800/PHE
${K}_{e}$(m)	8.896 × 10^−3^ ± 1.88 × 10^−3^
${k}_{M}$(m s^−1^)	2.5 × 10^−7^
${k}_{m}$(m s^−1^)	6.628 × 10^−8^ ± 1.33 × 10^−7^
${k}_{f}$(m s^−1^)	7.014 × 10^−6^ ± 1.76 × 10^−6^
${D}_{e}$(m^2^ s^−1^)	3.63 × 10^−12^
${D}_{s}$(m^2^ s^−1^)	9.37 × 10^−13^

The multi-compartment model predicts satisfactorily the sorption kinetics over the early times but over-estimates or sub-estimates the sorption evolution over long times (Fig. 12a). Such discrepancies could be attributed to inconsistencies of the model associated with (i) the mean pore concentrations over each particle, and parallel non-intersecting pores, in contrast with the radial variation of concentration, and the pore interconnectivity in actual structures; and (ii) the consideration of AC grains of uniform radius $\langle {r}_{g}\rangle$ despite the existence of a grain size distribution (Table 6). On the other hand, it is worth mentioning that instead of any phenomenological model (1st-order, 2nd-order, intraparticle-diffusion, etc.), a physical model was used to interconnect the measured characteristics of adsorbent with PHE sorption dynamics.

The average PHE concentration dissolved in meso- /macro-pores is quite low (Fig. 12c) and has negligible effect on the total sorption capacity, ${q}_{t}$, Eq. (11). At early times, PHE diffuses very fast through the outer film, and is adsorbed onto the external particle surface (Fig. 12b). As the PHE concentration in bulk decreases rapidly, the PHE concentration adsorbed on the external particle surface also decreases since it is at equilibrium with its concentration in the bulk (Fig. 12b). Gradually, PHE is transferred via molecular diffusion and adsorbed on the meso-/macro-pores, while surface diffusion transfers PHE molecules to the micro-pores (Fig. 12b). At late times, most of the adsorbed mass has been transferred to meso-/macro-porosity (Fig. 12b) while the PHE concentration adsorbed on the micro-pores might become discernible at very late times (Fig. 12b).

#### Sorption mechanisms

The adsorption of PAHs may be governed by three main mechanisms: (i) electron donor–acceptor interaction; (ii) formation of hydrogen bonding; and (iii) π-π interactions related to hydrophobic interactions (Esfandiar et al. 2021; Yuan et al. 2010). At the electron donor–acceptor mechanism, the carbonyl groups (C = O) at the surface of the adsorbent act as donors of electrons while the aromatic rings of the PAH act as acceptors. For this interaction, it is necessary that carbonyl groups are predominant. ATR-FTIR characterization revealed that all CWACs of the present study lost their lignocellulosic structure after CW pyrolysis, so that many functional groups including carbonyl (Fig. 6) were transformed into fully graphitic materials (Raman spectra, Fig. 7). The hydrogen bonding is established when the oxygen of the surface of the adsorbents is attached to water molecules. Water is adsorbed on oxygen-containing functional groups and the generated adsorption sites attract additional water molecules (Müller and Gubbins 1998). Based on this mechanism, the insertion of PHE into the porous structure probably would be prevented since polar groups are located mainly at pore entrances (Donnet 1982). The simulation of sorption dynamics with the multi-compartment model revealed that the process kinetics is governed by pore diffusion in the pore network of CWAC-NaOH-800, which was analyzed from N_2_ sorption isotherms and MIP data (Fig. 4; Table 3) and confirmed with SEM images (Fig. 5). In addition, as it has already been mentioned at the ATR-FTIR spectra, no functional groups are evident on the surface of CWAC-NaOH-800. Moreover, if CWAC-NaOH-800 exhibited polar behavior and preferential adsorption of water, it would contain a large amount of oxygen and present a hydrophilic nature (Ania et al. 2007). Furthermore, the abovementioned mechanisms would be active if the process was pH-dependent. However, the amount of PHE adsorbed onto CWAC-NaOH-800 was not sensitive to pH (Fig. 8; Fig. 9b), and the *ζ*-potential of CWAC-NaOH-800 was independent on pH, both indicating the absence of PHE electrostatic attraction on the adsorbent surface. The hydrophobic nature of CWAC-NaOH-800, like the majority of activated carbons (Gonçalves et al. 2010), favors the intense π–π interactions between the π-electrons of PHE aromatic rings and π-electrons of graphene layers, which seem to be the dominant sorption mechanism.

## Conclusions

The present work examines the potential to remove the recalcitrant pollutant phenanthrene (PHE) from water matrices with adsorption on low-cost agricultural waste and natural inorganic compounds: (i) untreated coffee waste (CW), and activated carbon produced from coffee waste (CWAC) pre-activated with NaOH or H_3_PO_4_, and (ii) untreated or treated with NaOH or H_3_PO_4_ diatomaceous earth. Based on their adsorption capacity, and pore structure characteristics, the most efficient adsorbent was the activated carbon CWAC-NaOH-800, produced with pre-treatment of coffee waste with NaOH, followed by pyrolysis at 800 °C. The high PHE sorption capacity of CWAC-NaOH-800, compared to the other adsorbents, was consistent with its high specific surface area (*S*_BET_ = 676.5 m^2^/g), total pore volume (*V*_MIP_ = 3.134 cm^3^/g), and well-developed meso- and macro-pore network. Parametric analysis revealed that PHE sorption on CWAC-NaOH-800 is independent of pH and reaches a maximum value of ~ 142 mg/g, as it is estimated from the Sips sorption isotherm model. The performance of CWAC-NaOH-800, compared to analogous adsorbents, developed earlier, was satisfactory towards the removal of PHE from water matrices. The PHE sorption dynamics was analyzed with the multi-compartment model that enabled the estimation of the mass transfer coefficients, and the quantification of the relative contribution of each compartment (external surface, meso-/macro-pores, micro-pores) to the total sorption. At late times, most of the adsorbed mass has been transferred to meso-/macro-porosity while the PHE concentration adsorbed on the micro-pores might become discernible at very late times. Accounting for the physicochemical properties of adsorbent, the insensitivity of PHE sorption capacity to pH, and hydrophobic graphene layers prevailing on the surface of CWAC-NaOH-800, it seems that the dominant sorption mechanism is the π–π interactions between PHE rings and CWAC-NaOH-800. The novel technology of cold atmospheric plasma (CAP) was applied for the regeneration of CWAC-NaOH-800 by using a DBD plasma microbubble reactor (CAP-bubbling). The experiment revealed the good reusability of the adsorbent and the recovery of sorption capacity, $\mathrm{REC}$, was found to be ~ 35.5%, exceeding the removal efficiency of the third adsorption cycle.

The new achievements of the present study are summarized briefly below.

- Activated carbon fabricated from coffee wastes with NaOH pre-treatment and pyrolysis at 800 °C (CWAC-NaOH-800) is a macro-porous adsorbent, which proved very suitable for the removal of PAHs from wastewater.
- The explicit correlation of the pore structure properties with the PHE sorption history and its spatial distribution over the various compartments of porosity prepares the bases for a cost-effective and fast method to assess the sorption capacity of activated carbon from pore space analysis and kinetic sorption tests.
- The adsorbent regeneration and reactivation of sorption sites by CAP seems well-promising, in the light of the following points: (i) The adsorbent CWAC-NaOH-800 could have been reused until full saturation, prior to CAP regeneration for obtaining a clear view of the regeneration mechanism from the recovery percentage; (ii) more regeneration cycles could be applied until full recovery of adsorbent; (iii) the conditions of the regeneration by CAP should be adapted to the adsorbent mass and its degree of saturation.

The present study could motivate a two-stage process for the remediation of PAH-polluted wastewater: (i) PAH sorption on low-cost adsorbents, appropriately fabricated from coffee wastes; (ii) regeneration of saturated adsorbent and reactivation of sorption sites by CAP. In this manner, the direct oxidation of pollutants and potential creation of toxic intermediate products in water might be avoided, and replaced by the repeated use of the regenerated adsorbent. When the two-step technology is scaled-up and applied at pilot scale under continuous or semi-batch conditions, a life cycle assessment could enable us to assess the cost-effectiveness and sustainability of such an approach, in terms of energy consumption and environmental fingerprint.

## Data Availability

The data that support this study will be shared on request to the corresponding author.
